# Endosome maturation is orchestrated by inside-out proton signaling through a Na^+^/H^+^ exchanger and pH-dependent Rab GTPase cycling

**DOI:** 10.21203/rs.3.rs-5515864/v1

**Published:** 2026-02-04

**Authors:** YouJin Lee, Qing Ouyang, Li Ma, Morgan Fleishman, Hasib Aamir Riaz, Michael Schmidt, Jeffrey L. Dupree, Anupam Mondal, Priyesh Mohanty, Jeetain Mittal, Oliver Beckstein, David G. Lambright, Eric M. Morrow

**Affiliations:** 1 Department of Molecular Biology, Cell Biology and Biochemistry, Brown University, Providence, RI, USA, 02912; 2 Center for Translational Neuroscience, Carney Institute for Brain Science and Warren Alpert Medical School, Brown University, Providence, RI, USA, 02912; 3 Department of Anatomy and Neurobiology, Virginia Commonwealth University, Richmond, VA, USA, 23284; 4 Research Service, McGuire Veterans Affairs Medical Center, Richmond, VA, USA, 23249; 5 Artie McFerrin Department of Chemical Engineering, Texas A&M University, College Station, TX, USA, 77843; 6 Department of Chemistry, Texas A&M University, College Station, TX, USA, 77843; 7 Interdisciplinary Graduate Program in Genetics and Genomics, Texas A&M University, College Station, TX, USA, 77843; 8 Department of Physics, Arizona State University, Tempe, AZ, USA, 85287; 9 Center for Biological Physics, Arizona State University, Tempe, AZ, USA, 85287; 10 Program in Molecular Medicine and Department of Biochemistry and Molecular Biotechnology, University of Massachusetts Chan Medical School, Worcester, MA, USA, 01605

## Abstract

Endosome maturation requires lumen acidification. Is progressive lumen acidification sensed by cytosolic-side molecules driving maturation? We show here that proton efflux through the endosomal Na^+^/H^+^ Exchanger (NHE6) activates the late endosome master regulator Rab7. Importantly, NHE6 is mutated in the childhood neurologic disorder Christianson Syndrome. We demonstrate that NHE6 interacts with the Rab7 GTPase-activating protein (GAP) TBC1D5 in a complex with Rab7 on the late endosome. This interaction and proton efflux are both required for Rab7 activation. TBC1D5 is potently inactivated with decreasing pH. A conserved histidine in the TBC1D5 GAP domain mediates pH-dependence. Furthermore, we show that neurons from mice engineered with a selective defect in NHE6 proton efflux exhibit blocked endosome maturation and disrupted Rab7 GTP-GDP cycling. In addition, knock-down of TBC1D5, thereby reducing Rab7 GAP activity, in NHE6 mutant neurons rescues Rab7 GTP-GDP cycling and endosome maturation. Finally, we present a biophysical model of proton signaling through acidic pH microdomains within the NHE6-TBC1D5-Rab7 protein complex upon endosome acidification. In conclusion, our studies provide evidence supporting a mechanism involving “inside-out” proton signaling, whereby lumen acidification drives endosome maturation through pH-dependent Rab GTPase cycling. Failure in this mechanism may have broad impact in neurodegenerative disease.

## Introduction

Endosomes mature from early to late endosomes, and eventually fuse with lysosomes^1^. Endosome maturation involves coordinated regulation of Rab protein activity on cytosolic-side, membrane surfaces, as well as progressive acidification of the endosomal lumen^1,2,3,4^. Rab proteins are small Ras-like GTPases, that are highly conserved from yeast to mammals^5,6^, and cycle between active GTP-bound and inactive GDP-bound states^7,8,9,10,11^. During trafficking, each intra-cellular compartment is associated with distinct Rab proteins and their effectors that control downstream functions. For example, Rab7 is localized to late endosomes and is a master regulator of late endosome functions, including trafficking and endosome-lysosome fusion^4,9^. Active GTP-bound Rab7 binds to effectors, such as Rab Interacting Lysosome Protein (RILP) on endosomal membranes^12,13^. Active GTP-bound Rab7 is deactivated by the Rab7 GTPase-activating protein (GAP) TBC1D5^14,15^. Inactive GDP-bound Rab7 no longer binds to these effectors and is extracted from the endosome membrane to the cytosol by Rab GDP dissociation inhibitor (GDI)^16,17,18,19,20,21,22,23^.

In addition to cycling of Rab GTPases, endosome maturation is also associated with gradual acidification of the endosome lumen^2,24^. In neurons, the endosomal lumen becomes progressively more acidic as endosomes mature and undergo retrograde transport from the distal projections toward the soma where they may fuse with lysosomes^13,25,26,27,28,29,30,31,32^. This acidification is mediated by the vacuolar ATPase (v-ATPase), which actively pumps protons into endosomes^24,33,34,35^. By contrast, endosomal Na^+^/H^+^ exchangers (NHEs), such as NHE6 which is abundant in neurons, permit proton efflux from endosomes. Long-standing questions in the field of endosome biology are: To what extent is endosome maturation driven by lumen acidification? What are the mechanisms whereby the cytosolic machinery governing endosome maturation senses lumen acidification? In this study, we demonstrate that endosomal NHE6 functions in “inside-out” proton signaling and links lumen acidification with Rab GTPase activity.

Importantly, loss-of-function mutations in NHE6 cause the X-linked neurological disorder Christianson Syndrome (CS). CS is characterized by postnatal microcephaly, intellectual disability, non-verbal status with autistic features, and involves neurodegeneration with motor abnormalities^36,37,38^. In our previous study, we generated an NHE6-null rat which exhibits early evidence of endosome and lysosome dysfunction, preceding prominent axonal loss^39^. However, despite the broad relevance of endolysosomal processes in both neurodevelopment and neurodegeneration^40,41,42^, the molecular mechanisms mediating pathogenesis in CS remain unclear. Our laboratory and others have shown, in a variety of cell types, that loss of NHE6 leads to over-acidified endosomes^31,43,44,45,46^, suggesting that NHE6 plays a role in regulation of intra-endosomal pH; however, the endogenous functions of endosomal NHEs remain poorly defined. We have previously demonstrated that NHE6-null neurons exhibit defects in endosome maturation, including impairments in endosome-lysosome fusion^25,31^. This endosome-lysosome fusion defect is conserved in the homologous yeast mutant Nhx1^47^.

In this study, we demonstrate that proton efflux through NHE6 is required to activate Rab7, thereby regulating late endosome maturation, linking endosome lumen acidification to Rab GTP-GDP cycling. We identify the Rab7 GAP, TBC1D5, as an NHE6 interactor protein that assembles in a complex on the endosome cytosolic leaflet with Rab7. We demonstrate that TBC1D5 is a pH sensor, and we pinpoint a conserved histidine within the TBC1D5 active site which mediates pH-dependent inactivation of GAP activity. In the absence of NHE6, TBC1D5 is hyperactive, resulting in impaired Rab7 GTP-GDP cycling and subsequent defects in late endosome axonal retrograde motility and endosome-lysosome fusion. To specifically define the role of NHE6-mediated proton efflux in Rab7 GTP-GDP cycling and endosome maturation, we generated an efflux-defective NHE6 mutant mouse (NHE6-ED). In neurons from this mouse, we find that NHE6-mediated proton efflux is required for Rab7 activation and proper endosome maturation. Furthermore, knockdown of the Rab7 GAP in NHE6-null and NHE6-ED neurons rescues defects in Rab7 GTP-GDP cycling and endosome maturation. Finally, we present a biophysical model for a new proton signaling mechanism whereby proton efflux through NHE6 regulates Rab7 activation by generating low pH microdomains in the NHE6/TBC1D5/Rab7 complex on the endosome leaflet. In summary, this study provides evidence for a new function for the CS protein NHE6 in signaling, using protons to drive endosome maturation in neurons. These results introduce inside-out proton signaling as a process regulating pH-dependent cytosolic proteins and a new fundamental mechanism in neurodegenerative disease.

## Results

### Loss of NHE6 leads to enlarged Rab7-positive late endosomes and decreased axonal retrograde motility.

**Fig. 1:**
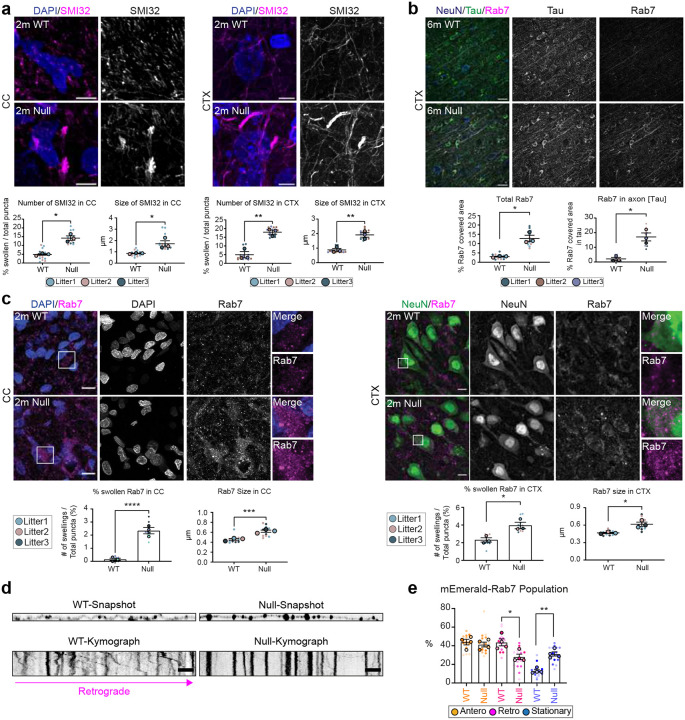
Rab7-positive late endosome defects in NHE6-null rats. **a,** Enlarged SMI32-stained (axonal damage marker, magenta) swellings in the corpus callosum (CC) and cortex (CTX) of NHE6-null rat brains and their littermate controls at 2 months. The number of SMI32-stained swellings were divided by the total number of SMI32 puncta. The average size of puncta was also quantified. Two-tailed unpaired t-test with Welch’s correction. Scale bar, 5 μm. **(a-c)** Means from each independent experiment (big dots, WT = 3, Null = 3 animals) overlay the entire dataset (small dots, 5 sections per each animal) and used for statistical analysis. Animals from same litters are color-coded. **b,** Accumulation of Rab7 late endosomes in the axons of NHE6-null rat cortex at 6 months. The percentage (%) of Rab7 area covered per image and within Tau-stained axons were quantified respectively. Two-tailed unpaired t-test with Welch’s correction. Scale bar, 20 μm. **c,** Enlarged Rab7 late endosomes in the CC and CTX of NHE6-null rats and their littermate controls at 2 months. White rectangles indicate magnified insets. The number of enlarged Rab7 (bigger than 1.5 μm) was divided by the total number of Rab7 puncta (%). The average size of Rab7 puncta was also measured. Two-tailed unpaired t-test with Welch’s correction. Scale bar, 10 μm. **d,** Representative snapshots and kymographs from WT and NHE6-null neurons show the motility of mEmerald-Rab7. Retrograde direction is indicated. Scale bar, 1 μm. **e,** Increased numbers of stationary mEmerald-Rab7 labelled endosomes in NHE6-null neurons. Decreased numbers of retrograde Rab7 endosomes were also detected in NHE6-null neurons. Anterograde, retrograde, and stationary endosomes were divided by the total number of mEmerald-Rab7 endosomes. Means from each animal (big dots, WT = 4, Null = 4 pups) overlay the entire dataset (small dots, n = 17–20 neurons from four independent experiments) and used for statistical analysis. Animals from same litters are color-coded. Ordinary one-way ANOVA with Tukey’s HSD. Data are represented as mean ± SEM. ****p < 0.0001, ***p < 0.001, **p < 0.01, and *p < 0.05.

NHE6 is abundantly expressed on endosomes in axons and dendrites^31,48^. We previously observed axonal pathology and axonal loss in aged NHE6-null rat brains at 12 months^39^. To investigate the impact of loss of NHE6 on neuronal processes preceding degeneration, we stained coronal sections from the corpus callosum (CC) and cortex (CTX) of NHE6-null rats and their littermate controls using an SMI32 antibody. SMI32 is known to indicate axonal damage by recognizing non-phosphorylated neurofilament heavy (NF-H) existing in neuronal axons^49,50,51^. At 2 months, we observed an increased size of SMI32 punctate and a greater number of enlarged SMI32-stained puncta in the CC and CTX in NHE6-null rats compared to those of wild-type (WT) rats (Fig. 1a). Over time, the intensity of SMI32 staining increased in NHE6-null rats compared to WT rats (Extended Data Fig. 1a). In addition, axonal degeneration was confirmed in NHE6-null rat brains as early as 2 months using electron microscopy (Extended Data Fig. 1b).

To evaluate the extent to which abnormal endosomes accumulated in swollen axons, we stained the cortex sections from WT and NHE6-null rats using markers for endosomes and axons (Tau). We observed an increased staining of total Rab7, a marker of late endosomes, and its staining within the axons in NHE6-null rat brains compared to WT (Fig. 1b). The number of swollen Rab7-positive endosomes (diameter larger than 1.5 μm) and average size were also quantified in the corpus callosum and cortex sections from WT and NHE6-null rat brains at 2 months (Fig. 1c). NHE6-null rats exhibited both a higher number and larger size of Rab7-positive structures in the corpus callosum and cortex when compared to WT rats. As NHE6 is also localized on early endosomes^31,44,52^, we stained sections from WT and NHE6-null rat brains with Rab5 (early endosomal marker). However, we did not detect swollen Rab5-positive endosomes or significant differences in the average size of Rab5-positive endosomes between WT and NHE6-null rats (Extended Data Fig. 2a).

Given our result of abnormal Rab7 late endosomes in NHE6-null brain, we set out to demonstrate that NHE6 is functioning in proton-efflux in late endosomes specifically. We loaded primary hippocampal neurons with fluorophore-conjugated dextrans for measuring luminal pH and quantified pH within endosomes tagged using a Rab7-RFP construct (Extended Data Fig. 3a). In control neurons, this method led to the measurement of intra-endosomal pH at 5.50 (± 0.086) which is as expected for late endosomes^53^. Notably, we found that the lumen pH in late endosomes in NHE6-null neurons was 5.15 (± 0.12), reflecting over-acidification of the intra-endosomal compartment with loss of NHE6. Thereby, these data provide support that NHE6 functions in proton efflux in late endosomes, consistent with prior data on the role of NHE6 throughout the endosome compartment in neurons^25,31,39^.

Previous studies have linked axonal loss to endolysosomal motility defects^49,54,55^. Given our observation of abnormal Rab7-positive endosomes, to investigate late endosome motility in axons, we transfected mEmerald-Rab7 into primary neurons from WT and NHE6-null rats (Fig. 1d). We confirmed that Rab7 endosomes bidirectionally moved in anterograde and retrograde directions (Fig. 1d)^56^. However, NHE6-null neurons have a higher number of stationary mEmerald-Rab7 and fewer retrogradely moving mEmerald-Rab7 endosomes in comparison to WT neurons (Fig. 1e). Also, the speed of both anterograde and retrograde Rab7 was faster in NHE6-null neurons than in WT neurons (Extended Data Fig. 3b). Furthermore, the size of mEmerald-Rab7 endosomes (Extended Data Fig. 3c, 3d, 3e) is larger in primary NHE6-null neurons compared to those in WT neurons. Notably, we also found that endogenous Rab7-positive endosomes were larger in NHE6-null neurons by staining for Rab7 (Extended Data Fig. 3f). Importantly, by contrast, we did not observe motility defects or size defects in mEmerald-Rab5 labelled endosomes in NHE6-null neurons (Extended Data Fig. 2b, 2c). In summary, our findings indicated that NHE6-null neurons exhibited an accumulation of enlarged Rab7 endosomes and late endosomal motility defects.

### Rab7 GTP-GDP cycling is disrupted in NHE6-null neurons.

**Fig. 2:**
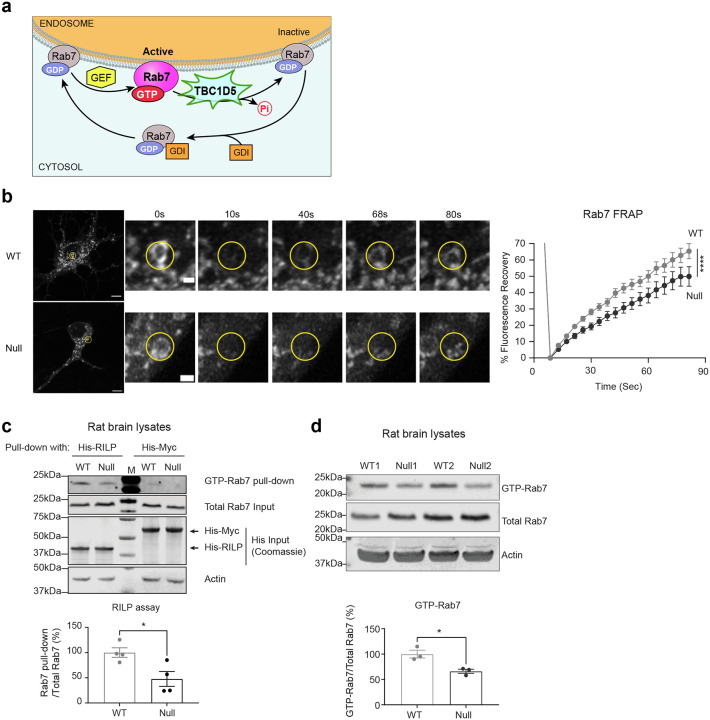
Rab7 GTP-GDP cycling is disrupted in NHE6-null rat brains. **a,** Schematic of GTP-GDP exchange cycle of Rab7. TBC1D5 is an GTPase activating protein (GAP) of Rab7. TBC1D5 promotes the hydrolysis of active, GTP-bound Rab7 to its inactive, GDP-bound form by stimulating GTP hydrolysis. Inactive Rab7 is then solubilized and extracted from membranes by GDP dissociation inhibitors (GDIs). Subsequently, guanine nucleotide exchange factors (GEFs) catalyze the exchange of GDP for GTP to reactivate Rab7. **b,** Representative images of fluorescence recovery after photobleaching (FRAP) assay with mEmerald-Rab7-transfected primary neurons at different time points. Slower recovery of mEmerald-Rab7 was detected from primary NHE6-null neurons compared to WT littermate neurons. Yellow circles indicate where photobleaching occurs and the fluorescence intensity was measured (WT = 4, Null = 4 pups, n = 35–37 neurons from four independent experiments). Non-linear mixed model was conducted. Scale bar, 5 μm. Scale bar for time course images, 0.1 μm. **c,** Reduced RILP-bound Rab7 (GTP-Rab7 pull-down) in NHE6-null rat brains at 2 months. Lysates from NHE6-null and WT littermate rat brains were incubated with His-RILP recombinant proteins or His-Myc (negative control) for the pull-down assay. Once samples were eluted, Rab7 was detected by western blot. Protein markers were loaded in the middle and labeled as “M”. The densitometry of GTP-Rab7 was divided by the total Rab7 for the quantification (4 rat brains for each genotype). Two-tailed unpaired t-test with Welch’s correction. **d,** Decreased GTP-bound Rab7 in NHE6-null rat brains at 2 months. Lysates from WT and NHE6-null littermate rat brains were incubated with GTP agarose to enrich GTP-bound protein fractions. Rab7 was detected in the GTP-enriched fraction by western blot. The densitometry of GTP-bound Rab7 was divided by the total Rab7 for the quantification (3 rat brains for each genotype). Two-tailed unpaired t-test with Welch’s correction was performed. Data are represented as mean ± SEM. ****p < 0.0001, ***p < 0.001, **p < 0.01, and *p < 0.05.

Rab7 is a small GTPase that cycles between membrane-associated, active GTP-bound and cytosolic, inactive GDP-bound states (Fig. 2a)^7,8,9,10,11^. The GTP-GDP cycle of Rab7 is required for late endosomal maturation and retrograde transport in neurons^13,57^. As more stationary Rab7 endosomes were detected in NHE6-null neurons (Fig. 1d and 1e), we hypothesized that defective GTP-GDP cycling of Rab7 disrupts the retrograde transport and leads to more stationary Rab7 endosomes. To test this hypothesis, we employed multiple complementary approaches. We first performed fluorescence recovery after photobleaching (FRAP) in primary WT and NHE6-null neurons transfected with mEmerald-Rab7 (Fig. 2b). Previous studies showed that the fluorescence recovery of Rab7 is altered by its association to the membrane and GTP-GDP exchange kinetics^58,59,60^. We bleached a small region of Rab7 in the soma and recorded the fluorescence recovery over time. Recovery of fluorescence occurs when photobleached mEmerald-Rab7 diffuses from membrane and is replaced by fresh mEmerald-Rab7 protein and delivered via RabGDI to the cytosol^16^. The recovery of mEmerald-Rab7 in primary WT neurons recovered to ~60 % within 80 seconds (Fig. 2b). In contrast, the recovery of mEmerald-Rab7 in primary NHE6-null neurons was slower and recovered to ~45 % over the same time course, indicating disrupted Rab7 GTP-GDP exchange dynamics. To determine whether this disrupted Rab7 GTP-GDP cycling is also present for endogenous Rab7 *in vivo*, we next performed a RILP pull-down assay using rat brain lysates (Fig. 2c), as active GTP-bound Rab7 preferentially binds to RILP^61,62^. We incubated lysates from WT and NHE6-null rat brains at 2 months with His-RILP probes. A lower amount of active Rab7, bound to His-RILP probes in the lysates, was detected in NHE6-null rat brains compared to those of WT brains. We did not observe changes in the total Rab7 protein levels between WT and NHE6-null rat brains. To further validate these findings, we repeated the RILP pull-down using the non-hydrolysable analogs GTPγS and GDPβS (Extended Data Fig. 4a). In WT lysates, GTPγS and GDPβS produced robust, opposing effects on Rab7, as WT maintains dynamic nucleotide exchange. In contrast, these differences were diminished in NHE6-null lysates. Consistently, a reduction in GTP-bound Rab7 was observed in NHE6-null brain lysates incubated with GTPγS. Lastly, we performed a GTP-agarose pull-down assay (Fig. 2d)^63,64^. The lysates from WT and NHE6-null rat brains were incubated with a GTP-agarose to enrich the pool of GTP-bound proteins and followed by detection with anti-Rab7 antibody. A lower amount of active Rab7 bound to GTP-agarose was found in NHE6-null rat brain lysates compared to WT lysates. Notably, regarding these two assays, we did not observe a significant difference in the amount of active Rab5 bound to His-RILP (Extended Data Fig. 2d), or active Rab5 bound to GTP-agarose (Extended Data Fig. 2e) in WT as compared to NHE6-null rats. Overall, these corroborative results (from the FRAP studies, the RILP assay, and GTP-agarose pull-down assay) indicate defective Rab7 GTP-GDP cycling in NHE6-null neurons.

### Increased co-localization of Rab7 with its GTPase-activating protein TBC1D5 in NHE6-null neurons.

**Fig. 3:**
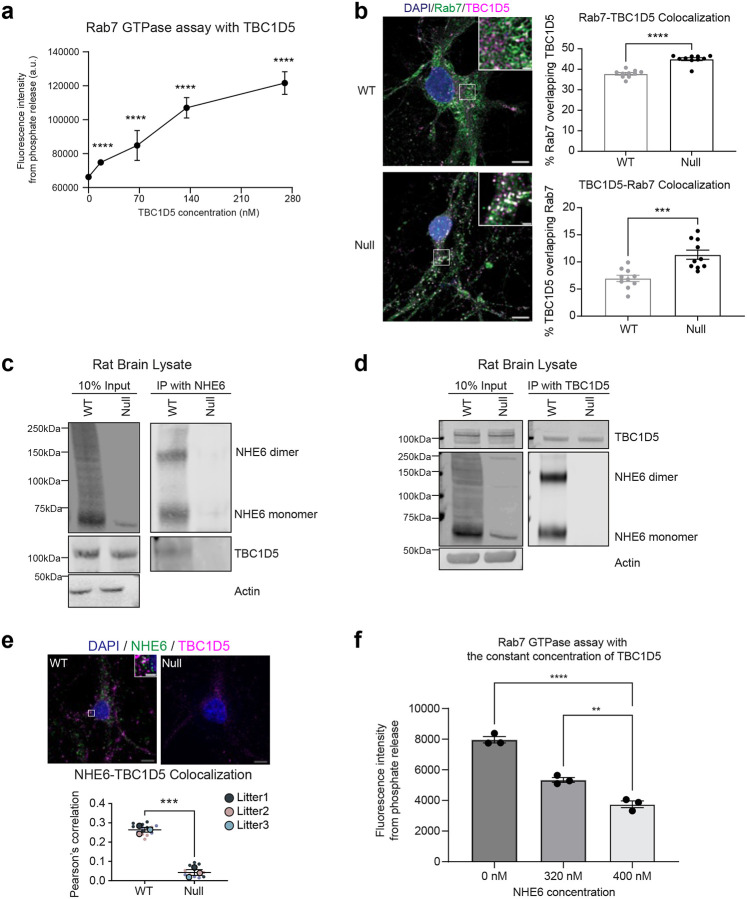
The interaction of NHE6 and TBC1D5 affects Rab7 GTPase activity. **a,** *In vitro* Rab7 GTPase assay shows that higher concentrations of TBC1D5 increased GTP hydrolysis of Rab7 measured by phosphate release. Pre-loaded GTP-Rab7 was incubated with various concentration of recombinant TBC1D5 for the luminescence measurement (n = 3 independent experiments). Ordinary one-way ANOVA with Dunnett’s test for multiple comparison was performed. **b,** Increased co-localization of Rab7 (green) and TBC1D5 (magenta) in NHE6-null neurons. The images were acquired using Nikon NSPARC. The quantifications show the Mander’s coefficient (M1: Rab7 overlapping TBC1D5, M2: TBC1D5 overlapping Rab7) from high-content imaging. Each dot indicates a mean of each pup (WT = 10, Null = 10 pups, n = 10,000–14,000 neurons imaged from each pup). Two-tailed unpaired t-test with Welch’s correction. **c,** Co-immunoprecipitation of NHE6 with TBC1D5 from WT and NHE6-null (negative control) rat brain lysates at 2 months shows the interaction of NHE6 with TBC1D5. **d,** Reciprocal immunoprecipitation from WT and NHE6-null littermate rat brain lysates at 2 months shows co-immunoprecipitation of TBC1D5 with NHE6. **e,** Representative images and the quantification of primary rat neurons show the co-localization of NHE6 (green) and TBC1D5 (magenta). Primary neurons were imaged using structured illumination microscopy (SIM). NHE6-null neurons were used as a negative control. A white box indicates the inset. The quantification presents Pearson’s correlation of NHE6 and TBC1D5 in WT and NHE6-null neurons. Means from each animal (big dots, WT = 3, Null = 3 pups) overlay the entire dataset (small dots, n = 13–15 neurons from three independent experiments) and used for statistical analysis. Two-tailed unpaired t-test with Welch’s correction was performed. Scale bar, 5 μm. Scale bar for the inset, 1 μm. **f,** *In vitro* Rab7 GTPase assay with increasing concentrations of NHE6. A constant concentration of GTP-Rab7 and TBC1D5 proteins (67.5 nM) was incubated with increasing concentration of NHE6 proteins to measure phosphate release. Higher concentrations of NHE6 decreased GTP hydrolysis of Rab7 mediated by TBC1D5. Ordinary one-way ANOVA with Tukey’s HSD. Three independent experiments were conducted. Data are represented as mean ± SEM. ****p < 0.0001, ***p < 0.001, **p < 0.01, and *p < 0.05.

Given our finding that the GTP-GDP cycling of Rab7 is disrupted in NHE6-null neurons (Fig. 2), we hypothesized that this is due to disrupted regulation of Rab7 activity by its GTPase-activating protein (GAP), TBC1D5^14,15^. Previous studies suggested that Nhx1, the NHE6 homologue in yeast, interacts with Gyp6, the TBC1D5 orthologue^47,65^. TBC1D5 is localized to late endosomes^15,62,66^, regulating GTP hydrolysis of GTP-bound Rab7 (as schematized in Fig. 2a). First, we tested whether TBC1D5 acts as a Rab7-specific GAP^14,15,67^. We measured Rab7 GTPase and Rab5 GTPase activities following incubation with varying concentrations of TBC1D5. TBC1D5 accelerates the GTP hydrolysis of Rab7, and consequently, more phosphates are released during this process. As expected, with increasing concentrations of TBC1D5, more phosphate released from Rab7 GTP hydrolysis was detected (Fig. 3a). However, TBC1D5 did not effectively hydrolyze GTP on Rab5 (Extended Data Fig. 2f).

We next investigated the localization of Rab7 and TBC1D5 in primary neurons from WT and NHE6-null rats using high-resolution microscopy with the Nikon Spatial Array Confocal (NSPARC) detector (Fig. 3b). Notably, NHE6-null neurons exhibited a markedly increased co-localization between Rab7 and TBC1D5 compared to WT neurons. To further assess their distribution, Rab7 activity was also assessed by subcellular fractionation, in which active, membrane-associated Rab7 was separated from the inactive, cytosolic pool. In NHE6-null brain lysates, cytosolic Rab7 levels were elevated compared to WT, accompanied by an increase in cytosolic TBC1D5 (Extended Data Fig. 4b).

### NHE6 interacts with the Rab7 GAP TBC1D5 at the cytosolic endosome membrane.

Previous studies using yeast two-hybrid assays identified an interaction between yeast Nhx1 (the yeast homolog of NHE6) and Gyp6 (the yeast orthologue of TBC1D5)^47,65^. To test whether this interaction is conserved in mammals, we repeated the yeast two-hybrid assay using the cytoplasmic tail of human NHE6 and full-length human TBC1D5 (Extended Data Fig. 4c). We detected an interaction between the cytoplasmic tail of human NHE6 and TBC1D5. To further examine this potential NHE6-TBC1D5 interaction in brain, reciprocal co-immunoprecipitation assays were conducted in lysates from both WT and NHE6-null rat brains at 2 months. Specifically, the lysates were immunoprecipitated with an anti-NHE6 antibody, and TBC1D5 was detected in the precipitates from WT rat brains (Fig. 3c). Reciprocal immunoprecipitations were also carried out using an anti-TBC1D5 antibody, which pulled down NHE6, confirming the TBC1D5 interaction with NHE6 in brain (Fig. 3d). We also observed high-resolution co-localization of NHE6 and TBC1D5 in primary WT neurons using structure illumination microscopy (SIM) super-resolution microscopy (Fig. 3e). Next, we examined the extent to which NHE6 protein interaction with TBC1D5 affects TBC1D5-mediated Rab7 GTPase activity *in vitro* (Fig. 3f). Recombinant Rab7 and TBC1D5 (67.5 nM) proteins were incubated with increasing concentrations of NHE6. Our data revealed that increasing concentration of NHE6 decreased the GTP hydrolysis of Rab7. These findings suggest that NHE6 biochemically hindered TBC1D5-mediated GTP hydrolysis of Rab7. Overall, our data suggest that the interaction between NHE6 and TBC1D5 limits TBC1D5 activity on Rab7.

### The proton efflux function of NHE6 regulates Rab7 activity.

We next asked if proton efflux via NHE6 regulates Rab7 activity. To test this, we used CRISPR/Cas9 to generate NHE6 efflux-defective (NHE6-ED) mice, wherein the exchanger function of NHE6 is inactive. We introduced two-point mutations (E235Q/D240N) in the cation exchanger domain—corresponding to human NHE6 mutations (E255Q/D260N) - known to block ion-proton transport^31,44^ (Extended Data Fig. 5a). We designed guide RNAs to substitute glutamic acid with glutamine (c.705G>C, p.E235Q) and aspartic acid with asparagine (c.720G>A and 722C>T, p.D240N), mimicking constructs previously studied extensively *in vitro* (Extended Data Fig. 5b)^31,68^. Sanger sequencing confirmed successful genome editing of *Slc9a6* in NHE6-ED mice (Extended Data Fig. 5c).

**Fig. 4:**
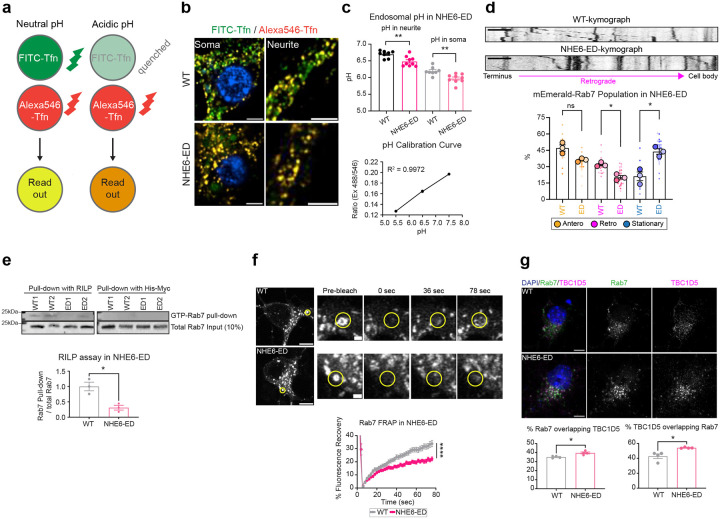
Regulation of Rab7 activity requires NHE6-mediated proton efflux. **a,** Schematic of the luminal pH measurement. Primary neurons are loaded with pH-sensitive, FITC-conjugated transferrin (Tfn) and pH-insensitive Alexa Fluor546-Tfn. More orange color indicates Tfn-positive endosomes with lower luminal pH. **b,** Representative images of the neurites and soma from WT and NHE6-ED neurons loaded with FITC-Tfn and Alexa Fluor546-Tfn. The decreased pH of endosomes in the neurites and soma of NHE6-ED neurons. Scale bar: 5 μm. **c,** Lower luminal pH of transferrin-positive endosomes in the neurites and soma of NHE6-ED neurons. The calibration curve was used to determine the luminal pH of transferrin-positive endosomes. Each dot indicates each pup (WT = 8, NHE6-ED = 9 pups, n = 10,000–14,000 neurons imaged from each pup). Two-tailed unpaired t-test with Welch’s correction. **d,** Representative kymographs show the motility of mEmerald-Rab7 endosomes in primary WT and NHE-ED neurons. Retrograde direction is indicated. Scale bar, 2 μm. Increased numbers of stationary mEmerald-Rab7 endosomes along with decreased retrograde numbers were detected in primary NHE6-ED neurons. Means from each animal (big dots, WT = 3, NHE6-ED = 3 pups) are overlay the entire dataset (small dots, n = 19–28 neurons from three independent experiments) and used for statistical analysis. Animals from same litters are color-coded. Ordinary one-way ANOVA with Tukey’s HSD. **e,** Decreased RILP-bound Rab7 (GTP-Rab7 pull-down) in NHE6-ED mouse brains at 2 months. Lysates from WT and NHE6-ED mouse brains were incubated with His-RILP recombinant proteins or His-Myc (negative control) for the pull-down assay. After elution, Rab7 was detected by western blot. The densitometry of GTP-Rab7 was divided by the total Rab7 (3 brains for each genotype). Two-tailed unpaired t-test with Welch’s correction. **f,** Slower FRAP recovery of mEmerald-Rab7 in NHE6-ED neurons. Yellow circles indicate where photobleaching occurs and the fluorescence intensity was measured (WT = 3, Null = 4 pups, n = 30–47 neurons from three independent experiments). Non-linear mixed model was conducted. Scale bar, 5 μm. Scale bar for time course images, 0.1 μm. **g,** Increased co-localization of Rab7 (green) and TBC1D5 (magenta) in NHE6-ED neurons. Quantification shows the Mander’s coefficient (M1: Rab7 overlapping TBC1D5, M2: TBC1D5 overlapping Rab7) from high-content imaging. Each dot indicates a mean of each animal (WT = 4, Null = 4 pups, n = 10,000–14,000 neurons imaged from each pup). Animals from same litters are color-coded. Two-tailed unpaired t-test with Welch’s correction. Scale bar, 5 μm. Data are represented as mean ± SEM. ****p < 0.0001, ***p < 0.001, **p < 0.01, and *p < 0.05.

We extensively studied the NHE6-ED mouse to confirm that NHE6 protein is expressed, trafficked normally, but impaired in proton efflux. Western blotting of brain lysates showed stable expression of both NHE6 monomer (~70 kDa) and dimer (~140 kDa) at levels comparable between WT and NHE6-ED (Extended Data Fig. 5d). Importantly, we measured the intra-endosomal pH in primary neurons from NHE6-ED and WT by fluorescent ratio imaging fluorescein isothiocyanate (FITC)-conjugated transferrin (Tfn; pH sensitive) to AlexaFluor-546-conjugated Tfn (pH insensitive) (Fig. 4a)^69^. Our data demonstrate decreased luminal pH in neuronal endosomes both in the soma and neurites in NHE6-ED neurons as compared to WT (Fig. 4b, c), indicating compromised proton efflux function of the NHE6-ED endosomes, akin to NHE6-null endosomes^31^. Immunostaining for Rab7 and NHE6 showed no differences in colocalization between WT and NHE6-ED (Extended Data Fig. 5e), but Rab7-positive endosomes were enlarged in NHE6-ED neurons (Extended Data Fig. 5f). Also importantly, co-immunoprecipitation confirmed NHE6-ED interaction with TBC1D5 (Extended Data Fig. 5g), supporting intact protein structure. Together, these results demonstrate that NHE6-ED localizes properly to endosomes but fails to mediate proton efflux, leading to endosomal over-acidification.

We investigated endosome transport and maturation in NHE6-ED neurons. We first investigated the extent to which motility of Rab7 endosomes is affected in NHE6-ED neurons. We monitored the motility of mEmerald-Rab7 endosomes in primary neurons from WT and NHE6-ED mice (Fig. 4d). NHE6-ED neurons have a higher number of stationary mEmerald-Rab7 endosomes and fewer retrograde mEmerald-Rab7 endosomes in comparison to WT neurons, akin to our prior observations in NHE6-null neurons (Fig. 1e). We did not observe differences in the speed of either anterograde or retrograde Rab7 endosomes (Extended Data Fig. 5h).

To determine the extent to which the proton efflux function of NHE6 affects GTP-GDP cycling of Rab7, we performed the RILP assay in WT and NHE6-ED brain lysates (Fig. 4e). Brain lysates were incubated with His-RILP and immunoblotted with anti-Rab7 antibody. A lower amount of active Rab7 bound to His-RILP probes in the lysates was detected in NHE6-ED mutant brains compared to those of WT brains, again akin to NHE6-null neurons. To further investigate the GTP-GDP cycling defects, we also performed the FRAP assay in primary WT and NHE6-ED neurons transfected with mEmerald-Rab7 (Fig. 4f). A small region of mEmerald-Rab7 was photobleached and its recovery was recorded over time. The recovery of mEmerald-Rab7 was slower in primary NHE6-ED neurons compared to those of primary WT neurons. Overall, neurons with the precise NHE6-ED mutation demonstrate similar defects to NHE6-null neurons.

Lastly, as increased co-localization of Rab7 with TBC1D5 was observed in NHE6-null neurons (Fig. 3b), we investigated the extent to which disrupted proton efflux caused by NHE6-ED affects TBC1D5 co-localization with Rab7. Primary WT and NHE6-ED neurons were stained with Rab7 and TBC1D5. The co-localization of Rab7 and TBC1D5 increased in NHE6-ED neurons compared to WT neurons (Fig. 4g), indicating that proton efflux via NHE6 is required for normal Rab7 interaction with TBC1D5 and that NHE6 proton efflux may reduce TBC1D5 activity on Rab7. In summary, our data show that proton efflux via NHE6 is required for proper Rab7 GTP-GDP cycling, regulating Rab7 localization with TBC1D5 and subsequently Rab7 activity.

### The GAP activity of TBC1D5 for Rab7 is pH-dependent.

Endosome maturation involves acidification of the endosome lumen. We hypothesized that TBC1D5 acts as a sensor that detects lumen acidification via proton efflux through NHE6 by adjusting TBC1D5 catalytic efficiency for acceleration of Rab7 GTP hydrolysis, i.e., converting GTP-bound to GDP-bound Rab7. In this way, NHE6 may function in inside-out signaling of endosome acidification, thereby promoting maturation by generating local decreases in pH on the cytoplasmic surface of the endosome.

**Fig. 5:**
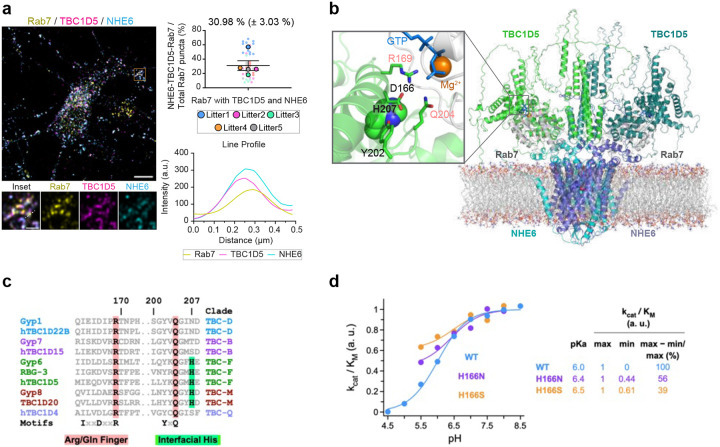
A conserved histidine in the GAP domain of TBC1D5 is critical for the pH sensing function. **a,** The co-localization of NHE6 (cyan), TBC1D5 (magenta), and Rab7 (yellow) in primary neurons at DIV7. Triple labelling is shown in white. An orange box indicates the inset. On average, 30.98 % (± 3.03%) of Rab7-punctae were co-stained with TBC1D5 and NHE6. Means from each animal (big dots, 5 WT pups) overlay the entire dataset (small dots, n = 40 neurons from five independent experiments). Intensity line profile shows the fluorescence intensity of each protein along a dashed line within the inset. The images were acquired using Nikon NSPARC. Data are represented as mean ± SEM. Scale bar, 5 μm. Scale bar for the inset, 1 μm. **b,** AlphaFold 3^70^ model showing a TBC1D5 dimer (upper top, green and turquoise) directly bound to the C-terminus of a NHE6 dimer (cyan and purple), which is embedded in a lipid bilayer. GTP-Rab7 (gray) is correctly positioned on the catalytic domain of TBC1D5 in this model. Note location of an interfacial histidine (H207) proximal to the invariant Arginine finger/aspartate and Glutamine finger/tyrosine. The AlphaFold 3 model was generated using the full-length NHE6 sequence. Note that N-terminal signal peptides was not present in the cryo-EM structure^71^ of NHE6 due to cleavage after Leu32 and also disordered structure between residues 32–73. **c,** Alignment of representative TBC domain orthologues and paralogues from the three major phylogenetic supergroups^105^. An interfacial histidine of TBC1D5 (H207) is conserved in orthologues (*C.elegans* RBG-3 and *Saccharomyces cerevisiae* Gyp6) and in one clade of paralogues (*S. cerevisiae* Gyp8 and human TBC1D20) but not in other paralogues. **d,** A conserved histidine located in the TBC domain is critical for the pH sensing function of TBC1D5. Catalytic efficiency (k_cat_/K_M_) of the *C.elegans* TBC1D5 orthologue is strongly dependent on pH. H166 of *C.elegans* TBC1D5 corresponds to H207 of human TBC1D5. The solid line in the graph represents a maximum likelihood-fitted model describing the pH dependence of one or more titratable groups. The data is normalized to the maximum value of the fitted model. In the WT, catalytic efficiency is strongly reduced with decreasing pH, whereas the magnitude of the pH effect is substantially diminished in the mutants. For WT, the fitted pK_a_ is 6.0 ± 0.11. Two mutants, H166N, and H166S, modestly increased pK_a_ values (H166N: 6.4 ± 0.31; H166S: 6.5 ± 0.57). WT reaches a maximum k_cat_/K_M_ of ~ 8700 ± 1100 M^−1^ s^−1^ with a large dynamic range of pH response, reflected in a maximum fractional difference (Max - Min / Max) ~100 %. H166N retains high maximal GAP activity (~ 4100 ± 300 M^−1^ s^−1^); however, the magnitude of the pH response is substantially diminished with a maximum fractional difference (Max - Min / Max) = ~56 %. H166S has substantially reduced maximum GAP activity (~ 880 ± 110 M^−1^ s^−1^) and the magnitude of the pH response is also substantially diminished with a maximum fractional difference (Max - Min / Max) = ~39%. (For WT, n = 2–5 experiments; for H166N and H166S, n = 4 experiments).

We first performed triple-immunofluorescence staining to investigate NHE6-TBC1D5-Rab7 co-localization in primary neurons using high-resolution microscopy with the NSPARC detector (Fig. 5a). We observe substantial co-localization of all three proteins together in primary neurons. On average, we found 30.98 % (± 3.03 %) of Rab7-positive late endosomes were co-stained with TBC1D5 and NHE6 (i.e., triple stained puncta). The intensity profile further confirms this co-localization within approximately 80 nm.

We used AlphaFold 3 to predict a structural model for a dimeric NHE6-TBC1D5-Rab7-GTP-Mg^2+^ complex with 2:2:2:2:2 stoichiometry (Fig. 5b)^70^. In this model, the TBC domain of TBC1D5 dimer binds to the C-terminal region of the NHE6 dimer^71^, which is embedded in a lipid bilayer. Within this interface, there is a conserved histidine (His 207) located proximal to these Arginine and Glutamine fingers, which is conserved in distant TBC1D5 orthologues and also in TBC1D20 orthologues, but not in other TBC domain paralogues (Fig. 5c).

To investigate the extent to which this conserved histidine contributes to the pH sensing function of TBC1D5, we first determined the catalytic efficiency (k_cat_/K_M_) of TBC1D5-catalyzed GTP hydrolysis on Rab7 over a broad range of pH values from 4.5–8.5. GTP hydrolysis was monitored by the change in intrinsic tryptophan fluorescence^18^ between GTP- and GDP-bound Rab7 in the absence or presence of the purified *C. elegans* TBC1D5 orthologue (RBG-3) (Fig. 5d, Extended Data Fig. 6a, and 6b). The data were acquired under single turnover conditions using the purified TBC and GTPase domains of TBC1D5 and Rab7 as described previously^72^. The k_cat_/K_M_ at each pH was determined (Extended Data Fig. 6b) directly from a simultaneous fit of the data over a range of TBC1D5 concentrations (see Methods). The k_cat_/K_M_ showed strong pH dependence, consistent with titration of one or more groups with similar pK_a_ (Fig. 5d). In contrast, Rab7’s intrinsic GTP hydrolysis rate is largely pH-independent without TBC1D5 (Extended Data Fig. 6a). TBC1D5 activity is thus highly pH sensitive—maximal activity at higher pH and minimal at lower pH—with a fitted pK_a_ (6.0 ± 0.11) matching that of free histidine (~6.04), implicating histidine protonation as a plausible mechanistic determinant.

To explore the contribution of the conserved histidine noted above to pH-dependence, we mutated the corresponding histidine in worm TBC1D5 to asparagine (H166N) or serine (H166S). Both asparagine and serine are common substitutions across diverse paralogues (Fig. 5c) and are in principle compatible with the structural environment of the conserved histidine in the AlphaFold 3 model as well as the crystal structure of the TBC1D20-Rab1 complex^73^. Substitutions to larger residues, including positively charged lysine or arginine, were not investigated since they rarely occur in paralogues and may be hard to interpret due to unavoidable van der Waals conflicts expected to prevent Rab7 binding or otherwise cause major structural rearrangement. To compare the pH effect on the catalytic efficiency of the mutants and WT proteins, the data was normalized to the maximum model value of k_cat_/K_M_ at high pH. At pH values below 5.5, aggregation of the mutant proteins interfered with reliable measurement of GAP activity. Accordingly, only data points obtained at pH values where no aggregation was observed are shown. Whereas decreasing pH drastically reduces the k_cat_/K_M_ of the WT protein, the magnitude of the effect is substantially diminished in both mutants (Fig. 5d), as evident from direct comparison of the observed k_cat_/K_M_ values in the pH range below 6.5 and further supported by maximum likelihood fitting with a pH dependence model function over the full range. Relative to the WT protein, there are two differences in fitted parameter values for the mutants (Fig. 5d): 1) a substantial reduction in the magnitude of the pH effect on k_cat_/K_M_, from ~100 % for WT to 56 % for H166N and 39 % for H166S; and 2) a modest increase in pK_a_ from 6.0 for WT to 6.4 ± 0.31 for H166N and 6.5 ± 0.57 for H166S. Because aggregation interfered with measurement of GAP activity for the mutants at the lowest pH values, we performed a rigorous statistical analysis of the potential impact of differences in the observable pH range and measurement noise on the certainty of maximum likelihood parameter estimation (see Extended Data Fig. 6c–e and Methods). Consistent with model-independent comparison of the observed k_cat_/K_M_ values, the results of this statistical analysis indicate that it is highly unlikely that the substantial differences in the magnitude of the pH effect are a consequence of either differences in the observable pH range or errors in the estimation of k_cat_/K_M_ values. The modest differences in pK_a_, on the other hand, have marginal statistical certainty. Together, the data and statistical analysis provide strong evidence that the two different conservative mutations diminish the magnitude of the pH effect, and further support an important role for the conserved histidine in enabling a robust response to pH changes such that TBC1D5 GAP activity approaches nil in the lower pH range.

Inside-out proton signaling requires that the local pH near the NHE6 proton-exit site and the conserved histidine in TBC1D5 be more acidic than the bulk cytoplasmic buffer (pH 7.2) for proton efflux to inactivate TBC1D5. We conducted mathematical modeling to investigate how pH may locally change between NHE6 and the cytosol. Using HOLLOW^74^ and networkX^75^, we estimated a plausible proton diffusion path from the NHE6 proton exit site to the pH-sensing histidine of TBC1D5 (H207), and onward into the cytosol^71^ (Fig. 5b, Extended Data Fig. 7a, and Supplementary Equation). Protons emitted from NHE6 will pass through cavities and tunnels that effectively represent a crowded protein-rich buffer^76,77,78^ – particularly when TBC1D5 is bound to NHE6 – and thereby the proton concentration will gradually decay with distance and reach the bulk cytosolic buffer solution at its distal end. This confined, approximately cylindrical, pathway (Extended Fig. 7a, colored in pink) represents the shortest path through the complex, with an estimated distance (*z*) of 7.5 nm between the NHE6 exit site and the pH-sensing histidine of TBC1D5. To estimate the local pH near this histidine residue, we solved the Laplace equation for protons appearing at the exit site of NHE6 and diffusing towards the cytosol, while incorporating limits on NHE6-driven proton export under physiological ion gradients^79,80^ due to thermodynamics and kinetics^81,82,83^ (Supplementary Equation). Under these conservative assumptions, the model predicts a gradual decay of proton concentration along the diffusion path, with a pronounced pH gradient maintained near the transporter exit. The local pH near the pH-sensing histidine was predicted to be at approximately pH 6.15 (Extended Data Fig. 7b), which is expected to reduce k_cat_/K_M_ by 40 % or greater based on the observed pH dependence of the WT protein (Fig. 5d). In summary, our cylindrical model predicts that proton efflux from NHE6 creates a local acidic microenvironment that would inactivate TBC1D5 based on our biochemical data.

### NHE6 activation of Rab7 requires interaction with TBC1D5

**Figure 6.**
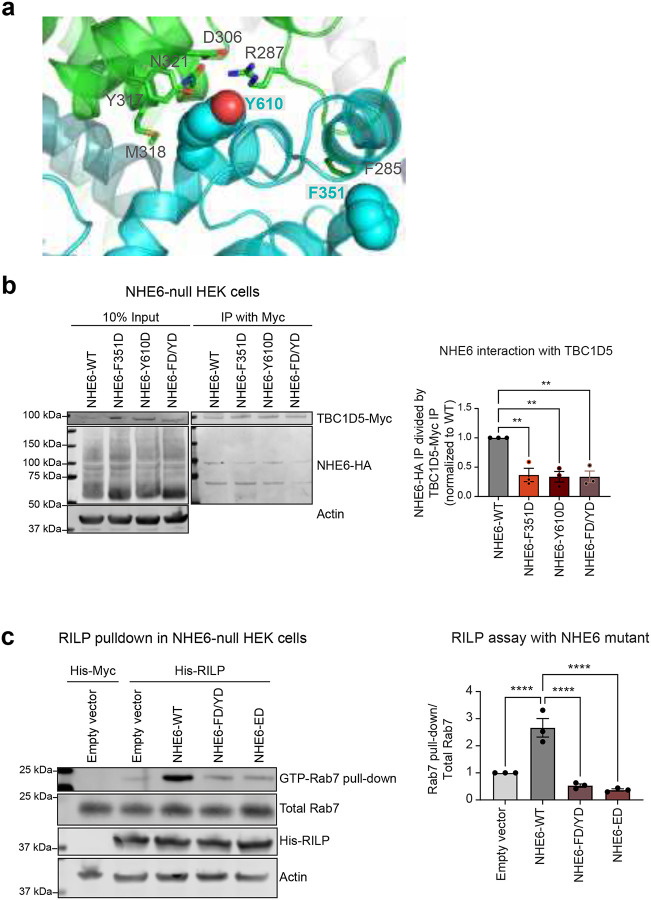
NHE6 activation of Rab7 requires interaction with TBC1D5 and proton efflux. **a,** AlphaFold 3^70^ model showing where NHE6 (cyan) interacts with TBC1D5 (green) via two residues (F351 and Y610 shown as spheres). **b,** Two residues of NHE6 are required for the interaction with TBC1D5. Co-immunoprecipitation of NHE6 mutants (F351D, Y610D, and F351D/Y610D) with TBC1D5 shows reduced interaction. NHE6-null HEK cells were transfected with NHE6-HA and TBC1D5-Myc constructs. Lysates were immunoprecipitated with anti-Myc antibodies and immunoblotted with anti-Myc (to detect TBC1D5) and anti-HA antibodies (to detect NHE6). The interaction was quantified by normalizing the immunoprecipitated HA signal to the immunoprecipitated Myc signal. Statistical analysis was performed using ordinary one-way ANOVA followed by Dunnett’s test for multiple comparisons (three independent experiments were performed). **c,** The RILP-bound Rab7 (GTP-Rab7 pull-down) was reduced in lysates expressing NHE6-FD/YD mutants. Lysates from NHE6-null HEK cells expressing either empty vector, NHE6-WT-HA, or NHE6-FD/YD-HA along with TBC1D5-Myc were incubated with His-RILP recombinant proteins for the RILP pull-down assay. Lysates from NHE6-null HEK cells expressing empty vector were incubated with His-Myc as a negative control. Once samples were eluted, Rab7 was detected by western blot. The densitometry of GTP-Rab7 was divided by the total Rab7 for the quantification (three independent experiments). Ordinary one-way ANOVA with Dunnett’s test for multiple comparisons. Data are represented as mean ± SEM. ****p < 0.0001, ***p < 0.001, **p < 0.01, and *p < 0.05.

Based on structural predictions from the AlphaFold 3 model (Fig. 5b) and computational modeling (Extended Data Fig. 4d)^84^, we identified two interacting residues i.e., F351 and Y610, of NHE6 (Fig. 6a). The F351 is found within the cytosolic loop between predicted transmembrane domains 7 and 8 of NHE6, and the Y610 is within the cytosolic tail. We generated single (F351D, or Y610D) and double (F351D/Y610D, referred to as FD/YD) mutants and performed co-immunoprecipitation experiments. All NHE6 mutants showed a strong reduction in interaction with TBC1D5 compared to wild-type NHE6 (Fig. 6b), supporting the direct interaction between NHE6 and TBC1D5.

We next conducted an experiment to demonstrate the importance of the NHE6-TBC1D5 interaction in GTP-GDP cycling of Rab7 (Fig. 6c). We expressed either empty vector, NHE6-WT-HA, or NHE6-FD/YD-HA in NHE6-null HEK cells and thereafter assessed active Rab7-GTP using the RILP assay. Importantly, expression of NHE6-WT restored levels of active, GTP-bound Rab7 compared to the empty vector, demonstrating the NHE6 is sufficient to activate Rab7. By contrast, the NHE6-FD/YD-HA mutant constructs were unable to activate Rab7, providing support that the interaction between NHE6 and TBC1D5 is required for the NHE6-mediated activation of Rab7. Furthermore, we demonstrate that the NHE6-mediated activation of Rab7 required proton efflux, as the NHE6-ED construct failed to activate Rab7. Together, our findings support a model in which TBC1D5 functions as a pH sensor that detects local pH changes driven by NHE6-mediated proton efflux. Through interaction with NHE6, TBC1D5 activity is modulated by NHE6 proton efflux promoting activation of Rab7.

### Knock-down of the Rab7 GAP TBC1D5 rescues endosomal phenotypes in NHE6 mutant neurons.

**Fig. 7:**
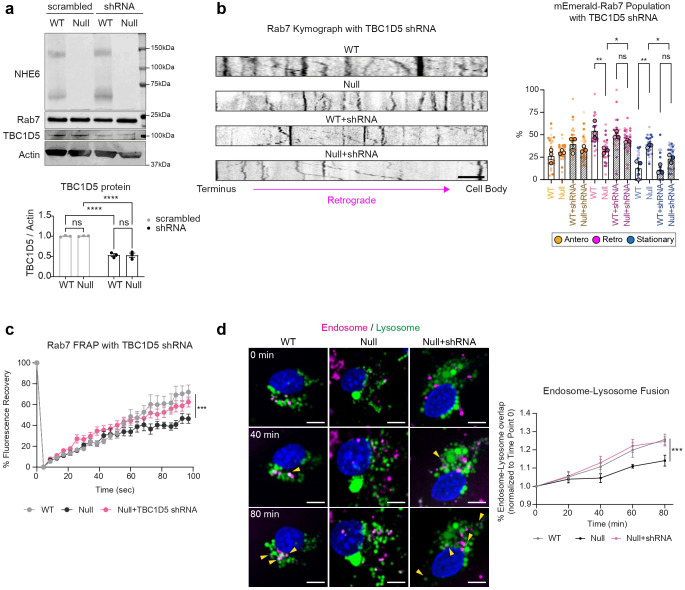
Knock-down of the Rab7 GAP TBC1D5 rescues endosomal phenotypes in NHE6-null rat neurons. **a,** Reduced protein expression of TBC1D5 in primary littermate WT and NHE6-null rat neurons, with lentiviral transductions of anti-TBC1D5 shRNA, as compared to scrambled shRNA control transductions The expression of NHE6 and Rab7 was not affected by the shRNA transduction (three independent experiments). Two-way ANOVA with Fisher’s LSD test was performed. **b,** Representative kymographs from primary littermate WT and NHE6-null neurons with scrambled or TBC1D5 shRNA show the motility of mEmerald-Rab7 endosomes. Retrograde direction is indicated. Scale bar, 1 μm. The number of retrograde Rab7 endosomes in primary NHE6-null neurons transduced with TBC1D5 shRNA was increased compared to NHE6-null neurons transduced with scrambled shRNA. Also, the number of stationary Rab7 endosomes was decreased in primary NHE6-null neurons transduced with TBC1D5 shRNA. Means from each animal (big dots, WT = 3, Null = 3 pups) are overlay the entire dataset (small dots, n = 21–31 neurons from three independent experiments) and used for statistical analysis. Animals from same litters are color-coded. Ordinary one-way ANOVA with Tukey’s HSD was performed. **c,** The FRAP recovery rate of mEmerald-Rab7 in primary NHE6-null neurons transduced with TBC1D5 shRNA exhibited significant increase relative to NHE6-null neurons with scrambled shRNA. The recovery rate of mEmerald-Rab7 in NHE6-null neurons transduced with TBC1D5 shRNA was comparable to WT neurons (6 pups in total for each genotype, n = 20–35 neurons from 3 experiments). Non-linear mixed model was conducted. **d,** The increased endosomes-lysosomes fusion in primary NHE6-null neurons transduced with TBC1D5 shRNA. Shown are still images at different time points from live imaging of the endosome-lysosome fusion assay on primary littermate WT and NHE6-null neurons transduced with scrambled or TBC1D5 shRNA at different time points. The level of endosome-lysosome fusion is presented as the percentage fold change in overlap between the endosome label (magenta) and the lysosome label (green), from time point 0 for the same animal. Yellow arrows indicate the fusion events. (WT = 5, Null = 5 pups, n = 10,000–14,000 neurons imaged from each pup). Linear mixed model was conducted. Scale bar, 5 μm. Data are represented as mean ± SEM. ****p < 0.0001, ***p < 0.001, **p < 0.01, and *p < 0.05.

Our data support the idea that in NHE6 mutant neurons TBC1D5 is over-active leading to diminished active Rab7. To determine the extent to which decreasing TBC1D5 activity on Rab7 in NHE6 mutant neurons rescues Rab7 defects in late endosomes maturation, we established a lentiviral system for acute knock-down of TBC1D5 levels. We transduced TBC1D5 shRNA lentivirus into primary WT and NHE6-null neurons at 2 days *in vitro* (DIV) and confirmed the decreased expression of TBC1D5 protein by western blot (Fig. 7a) and immunostaining with an anti-TBC1D5 antibody (Extended Data Fig. 8). We then monitored the motility of mEmerald-Rab7 endosomes in primary WT and NHE6-null neurons transduced either with scrambled shRNA or TBC1D5 shRNA (Fig. 7b). NHE6-null neurons transduced with scrambled shRNA displayed fewer retrograde Rab7 endosomes and a greater number of stationary Rab7 endosomes compared to WT neurons, as we observed previously in non-transduced neurons (Fig. 1e). However, transduction of TBC1D5 shRNA lentivirus in NHE6-null neurons reversed these phenotypes. Specifically, transduction with TBC1D5 shRNA in NHE6-null neurons significantly increased the number of retrograde Rab7 endosomes while decreasing the number of stationary Rab7 endosomes compared to NHE6-null neurons transduced with scrambled shRNA. We also performed the FRAP assay in primary WT and NHE6-null neurons transduced with scrambled or TBC1D5 shRNA (Fig. 7c). The recovery of Rab7 was significantly increased in primary NHE6-null neurons transduced with TBC1D5 shRNA, compared to primary NHE6-null neurons transduced with scrambled shRNA. Lastly, we had previously shown that endosome-lysosome fusion, a key step in endosome maturation, was impaired in NHE6-null neurons^25^. Furthermore, TBC1D5 plays a key role in endosomal fusion with lysosomes^62^. Late endosome fusion with lysosomes was monitored in TBC1D5 shRNA transduced neurons (Fig. 7d). The overlap of endosomes with lysosomes, signifying endosome-lysosome fusion, was significantly increased in primary NHE6-null neurons transduced with TBC1D5 shRNA, as compared to NHE6-null neurons with scrambled shRNA. To further test whether the knock-down of TBC1D5 rescues the Rab7 endosomal defects in NHE6-ED neurons, we conducted rescue experiments in primary NHE6-ED mouse neurons using TBC1D5 shRNA (Extended Data Fig. 9). Live-cell imaging of mEmerald-Rab7 revealed that the impaired motility observed in NHE6-ED neurons was rescued upon TBC1D5 knockdown (Extended Data Fig. 9a). Also, the FRAP assay showed increased recovery of mEmerald-Rab7 signal in TBC1D5 shRNA–transduced NHE6-ED neurons, indicating restored Rab7 dynamics (Extended Data Fig. 9b). Finally, endosome–lysosome fusion events were significantly increased following TBC1D5 knockdown in NHE6-ED neurons (Extended Data Fig. 9c), further supporting the functional relevance of TBC1D5 over-activation in the absence of NHE6-mediated proton efflux in the NHE6-ED cells. Overall, these data indicate knock-down of TBC1D5 rescues Rab7 functions in NHE6-null and NHE6-ED neurons, demonstrating that overactive TBC1D5 and dysregulated Rab7 are linked to endosome and motility defects in the absence of NHE6 function.

## Discussion

**Fig. 8:**
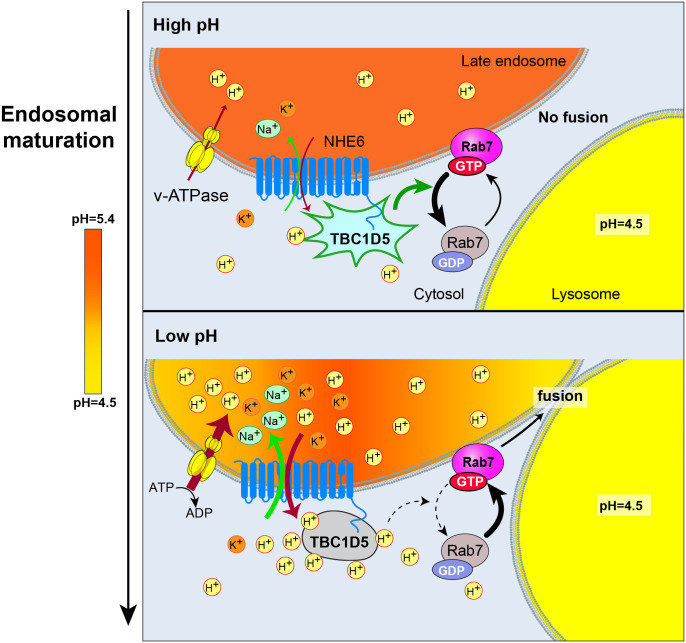
Proposed mechanisms by which endosome lumen acidification regulates Rab7 activity and endosome maturation, a process mediated by proton efflux through the Christianson Syndrome protein NHE6 and the pH-dependent Rab7 GAP TBC1D5. During endosome maturation, the v-ATPase pumps protons into the endosomal lumen. Early in endosome maturation, as the proton concentration is relatively lower (higher pH) **i),** proton efflux through NHE6 is limited, and TBC1D5 is highly active, accelerating Rab7-GTP hydrolysis. This promotes conversion to inactive Rab7-GDP. Consequently, late endosomes are not efficiently fused with lysosomes. As endosomes mature, the v-ATPase more actively pumps protons into the endosomal lumen. **ii),** During this phase, as proton concentration is higher (lower pH), NHE6 leaks protons from late endosomes. As late endosomes gradually acidify, the cytoplasmic microenvironment adjacent to NHE6 on late endosomes is progressively acidified. This change in local pH inactivates TBC1D5 by protonation of His207 in the GAP domain. The GAP activity of TBC1D5 on Rab7 is thereby inhibited, and as a result, Rab7 is converted to the active GTP-bound form, driving a molecular cascade leading late endosomes to fuse with lysosomes.

We show here that two classic processes in endosome maturation, progressive lumen acidification and Rab GTPase activity, are mechanistically linked. “Inside-out” proton signaling, whereby lumen acidification governs cytoplasmic-side molecular machinery, has been previously hypothesized but few mechanisms have been defined^1,35,85,86,87,88^. We provide evidence that proton efflux, mediated by a Na^+^/H^+^ exchanger, acts as an inside-out signal that coordinates lumen acidification and Rab7 regulation. We propose that this process operates through a pH-dependent Rab GAP that interacts with NHE6 in a complex with Rab7. Thereby, we identify a new function of endosomal NHEs, and the CS protein NHE6 specifically, in proton signaling. This mechanism likely functions downstream of Rab conversion^19,20,21,89,90^, potentially as a brake on Rab7-GTP accumulation until lumen acidification reaches the optimal pH range. Figure 8 illustrates our model in which NHE6-mediated proton efflux regulates Rab7 by inactivating the Rab7 GAP TBC1D5 in a pH-dependent manner to orchestrate endosome maturation. Our study also shows how perturbation of this process causes neurodegeneration, such as in Christianson Syndrome (CS).

Our experiments show that proton efflux through NHE6 is necessary for proper Rab7 cycling. We developed a new NHE6 exchanger-deficient mouse model (NHE6-ED). In this mouse, the NHE6-ED protein is endogenously expressed, localizes to endosomes and binds TBC1D5; however, as predicted, the endosome is over-acidified, reflecting the defect in proton efflux, and importantly, active GTP-bound Rab7 is reduced, and endosome motility and endosome-lysosome fusion are impaired, all akin to the NHE6-null. These phenotypes are rescued by TBC1D5 knockdown. Overall, these results provide strong evidence that in the absence of NHE6-mediated proton efflux TBC1D5 is over-active.

Our experiments also support a mechanism whereby an interaction of TBC1D5 with cytosolic domains of NHE6 regulates endosome maturation. Our AlphaFold 3 modeling predicts that the dyad-symmetric TBC1D5 dimer is positioned at the cytosolic surface of the NHE6 dimer, which is suited for proton sensing. Our structural and biochemical analyses further reveal that two residues - F351 in the cytosolic loop between transmembrane domain (TM7) and TM8, and Y610 in the C-terminal tail of NHE6 - are essential for TBC1D5 binding. Interestingly, a recent cryo-EM study revealed that the cytoplasmic tail (involving residues in the region of Y610) interacts with K350 in NHE6 through hydrogen bonds^71^. A similar interaction has been observed in NHE9, whereby this interaction has been proposed to be involved in auto-inhibition of cation exchange^91^. Thereby, binding of TBC1D5 at the proposed residues may serve to dislodge the auto-inhibition by the cytoplasmic tail and activate NHE6. Furthermore, protonation of TBC1D5 inactivates TBC1D5 activity and may introduce positive charge that facilitates association with membrane lipids and stabilizes this juxta-membrane association with NHE6^35,71,88,92^.

Importantly, our biochemical studies support the idea that a conserved histidine in the TBC domain acts as a major pH sensor through which the GAP is inactivated upon acidification. Previous studies in yeast demonstrated that Nhx1, the NHE6 homologue, interacts with the TBC1D5 orthologue Gyp6, supporting the evolutionary conservation of this functional interaction^47,65^. Among the 19 histidines in the TBC domain, four are conserved in the worm TBC1D5 orthologue, highlighting their potential functional importance. The key interfacial histidine is positioned adjacent to the catalytic Arg and Gln fingers and is conserved across other TBC1D5 and TBC1D20 orthologues^73^, highlighting its evolutionary and functional significance. It is important to note that histidine mutants still retain some pH-dependence, albeit to a much lesser extent than the wild-type protein (between ~39 to 56 %). It is possible that other conserved histidines outside of the interface also contribute to the pH sensing^93^. Those histidines may have modestly higher pK_a_ values (~6.4–6.5) compared to H207 (~6.0) (H207 of human TBC1D5 corresponds to H166 of *C.elegans* TBC1D5), although the statistical significance of the observed pK_a_ differences in the mutagenesis experiments is marginal. Our mathematical modeling further supports the formation of a localized pH gradient, where protons diffuse through the protein complex and dissipate into bulk cytosol at the tunnel end. Thermodynamic analysis of NHE6 transport under physiological ion gradients (Supplementary Equation)^79,80,94^ suggests that such proton efflux is energetically feasible and could generate a local pH environment substantially more acidic than the surrounding cytosol. Similar to our model, a very recent study also demonstrates that this nanometer-scale, acidic microdomain can persist on the cytosolic, juxta-membrane surface of lysosomes through proton efflux, without altering the bulk cytosolic pH^87^. Importantly, this localized pH reduction is predicted to be sufficient to alter the protonation status of the pH-sensing histidine in TBC1D5, providing mechanistic insight for pH-dependent regulation of its GAP activity. Notably, our modeling further indicates that potassium gradients are energetically more favorable than sodium gradients in driving proton efflux through NHE6^79^. Consistent with this observation, NHE6 and its yeast orthologue Nhx1^45^ have been reported to transport K^+^ in addition to Na^+^. This suggests that K^+^-dependent transport may play a role in creating the local pH microenvironment.

Our simple model highlights how proton efflux from NHE6 can create an acidic microenvironment within the complex. Future work determining proton leakage routes and kinetics within the NHE6-TBC1D5-Rab7 complex will help to refine our current model. Together, these findings suggest that NHE6-generated proton gradients regulate Rab7 cycling through histidine protonation of TBC1D5. Currently, there are no available pH sensors targeted to the endosomal pathway that can be used to measure the cytoplasmic juxta membrane pH at the required spatial and temporal resolution, despite recent developments in genetically encoded pH indicators^80,89,95,96^. A recent study has reported DNA nanodevices to monitor the cytoplasmic juxta membrane pH on the lysosomes^87^. However, this method has not been applied to endosomal compartments. Future studies will require high-resolution methods and rapid imaging speeds to directly visualize the dynamics of these local proton microdomains on endosomes^71,81,83,97,98^.

Loss-of-function mutations in NHE6 cause the X-linked neurological disorder Christianson syndrome (CS). CS patients display developmental delay, postnatal microcephaly, absent speech, progressive ataxia and epilepsy along with neurodegenerative disease^37,99,100^. There is prominent axonal pathology in both human postmortem and animal models^39,101^. Disrupted motility, and accumulation of enlarged and as well as clusters of endolysosomes have been implicated in the pathogenesis of other neurodegenerative disorders including Alzheimer’s disease^28,49,54,102^. In our NHE6-null and NHE6-ED neurons, we observed disrupted motility and the accumulation of enlarged Rab7-positive endosomes. Interesting, prior studies of the Nhx1 yeast have also observed swollen pre-vacuoles, equivalent to the late endosomes, and characteristic of the Type E yeast VPS mutants^47^. In previous studies in the NHE6-null rat brain, we have shown that the accumulation of enlarged endolysosomes preceded autophagosomes dysfunction^39^. In that study, we also observed that gliosis, axonal loss, neuronal loss and tau deposition occur in NHE6-null rat brains. The Rab7 defects may cause defects in axonal and dendritic trafficking, leading to cascading downstream effects during aging and neurodegeneration^64,103^.

Endosomal maturation involves a multitude of sequential events including luminal acidification and recruitment of molecular components. Previous studies showed that the Rab5-to-Rab7 conversion on the endosomal membrane promotes Rab7 activation^21^ and luminal acidification^89^. However, how lumen acidification and Rab activation are coordinated, and how lumen acidification drives cytosolic mechanisms are important questions. We speculate that the sharp drop in luminal pH during the early-to-late endosome transition modulates NHE6 activity and conformational change^35,81,83,97,104^, which in turn regulates TBC1D5 interactions with NHE6 and promotes GTP-bound Rab7 accumulation on endosomal membranes. Future work may identify additional Rab GAPs or GEFs whose activity is pH-sensitive, as well as other endosomal NHEs and downstream targets involved in proton-mediated signaling. Such discoveries will further illuminate a conceptually new mechanism in proton signaling and endosomal transport and its implication in basic biology and disease.

## Methods

### Rats and mice

All animal work was conducted under the guidelines of the Center for Animal Resources and Education (CARE) at Brown University with a protocol (IACUC 21-09-0012), approved by the Institutional Animal Care and Use Committee (IACUC). All experimental procedures were consistent with the US National Institutes of Health Guide for the Care and Use of Laboratory Animals (National Research Council, 8th edition). Animals were kept at a 12/12-hour light/dark cycle with ad libitum access to food and water. As NHE6 is located on the X chromosome, only male rats were used for this study. Heterozygous females were crossed with wild-type males for breeding. Tails from rats and mice were clipped and externally genotyped (Transnetyx, Cordova TN). NHE6-null rats were maintained on the Sprague-Dawley background and originally described in our previous study^39^. Age of animals used for this study were indicated in each figure and/or figure legends. A mouse model of human E255Q/D260N variant (mouse E235Q/D240D) was generated for this study. This line is referred to as NHE6 efflux-defective (NHE6-ED) mutant line. Mouse lines were generated using CRISPR/Cas9 genome editing (Mouse Transgenic and Gene Targeting Facility of Brown University) and are described for the first time herein. We used a guide RNA (5’- GGAAAGTGTCCTCAATGACG-3’) to generate both mutations. Mouse lines were generated on the C57BL/6J mouse background. One male founder and two female founders were generated and used for breeding. Studies were conducted in offspring from each of the different founders. Targeting of constructs and presence of mutations were confirmed by PCR genotyping and Sanger sequencing (Extended Data Fig. 4C). The following primers were used for PCR genotyping: forward primer (5’- AGCTGTGGAGGGATATGTGC-3’) and reverse primer (5’- TCAGAGCAGGGCAGAAAGAC-3’). The expression of protein was confirmed by western blot (Extended Data. 5d).

### Tissue preparation for staining and protein assays

For immunofluorescence staining, animals were anesthetized using pentobarbital (100 mg/kg). Animals were transcardially perfused using phosphate-buffered saline (PBS) followed by 3.7 % formaldehyde (Sigma-Aldrich). Following cardiac perfusion, the brain was stored in 3.7 % formaldehyde fixative at 4 °C overnight. For immunofluorescence, the brain was transferred to 30% sucrose solution at 4 °C and stored until sinking. Frozen brain samples were coronally sectioned at 50 μm thickness on a sliding microtome (Thermo Scientific #HM430) at −20 °C. Sections were serially collected and stored in 24-well plates containing a cryoprotectant solution (30% sucrose, 30% ethylene glycol, 1% Polyvinyl-pyrrolidone in PBS) at −80 °C until use.

For protein extraction, wild-type and NHE6-null rodents were euthanized with CO2 and brains were removed, dissected and cut in half. Each hemisphere was weighed, snap frozen and homogenized for protein extraction in NP-40 lysis buffer (Thermo Scientific # J60766.AP).

### Stereological sectioning and analysis

Anatomical brain regions of interest (ROI) were confirmed according to the rat brain atlas^106^. Three to five sequential coronal sections 300 μm apart between bregma −1.88 mm to −6.72 mm were taken per each animal. All procedures including staining, image acquisition, and analysis were performed blind to genotype and age. Only male animals were used.

All the quantification was performed by an investigator using Fiji ImageJ who was blinded to genotype and age of animals. For counting the number and size of SMI32 or Rab7, five serial coronal sections 300 μm apart from WT and NHE6-null rats were taken for staining and analysis. Images for each section were randomly taken within the defined ROI, i.e., CC and CA1 using 60x (NA 1.42) oil immersion objective (Olympus FV3000). The number and size of SMI32 per each image were manually counted. Also, the % of Rab7 covered area per each image was quantified. To quantify % of Rab7 covered area within axon, masks from Tau-stained images were created. If Rab7 puncta diameter size is larger than 1.5 μm, we defined them as swollen/enlarged Rab7. The number of swollen Rab7 puncta was divided by the total puncta number to present the percentage (%). For quantifying the Rab7 presence in the Tau-stained axons, we generated a mask from Rab7 staining and overlaid the Rab7 mask to tau staining in Fiji ImageJ. The intensity of Rab7 and % of Rab7 covered area in this mask were only counted.

All tissue sections were imaged under a confocal laser scanning microscope (Olympus FV3000) with the same imaging settings between control and mutant animals. Mutant and control images were equally adjusted in Fiji ImageJ software for brightness and contrast to ensure accurate data analysis. All data analysis was performed by someone blind to the genotypes and age of the animals using Fiji ImageJ.

### Primary neuronal culture

Hippocampi were dissected from P0-P1 rats or mice dissociated with papain dissociation kit (Worthington #LK003150) as previously described^31^. Only male rats and mice and their wild-type littermate controls were cultured for this study. Briefly, the brains were removed from the skull, and hippocampi were dissected on ice in pre-chilled Hank’s balanced salt solution (HBSS; Invitrogen #14025076). All dishes were pre-coated with 0.1 mg/mL of Poly-D-lysine (PDL; Sigma #A3890401) for overnight at 37 °C. Cells were cultured in Neurobasal-A media (Invitrogen #10888022) supplemented with 2 % B27 (Invitrogen #17504044), 1 % glutamax (Invitrogen # 35050061), and penicillin/streptomycin. Cells were seeded at densities of 28,000 cells per well in the 96-well plate (Perkin Elmer #6055302) for high-throughput imaging. 120,000 cells per dish were seeded for #1.5 14 mm glass bottom dishes (Cellvis #D35-14-1.5-N) for live-imaging. For immunofluorescence, 40,000 cells were seeded on each #1.5 12 mm PDL-coated German glass cover slips (neuVitro #GG-12–1.5-PDL) and placed in 24-well plates. 1.2×10^6^ cells per well were seeded for 6-well plate for western blotting. For shRNA lentivirus transduction, scrambled shRNA and TBC1D5 shRNA (MOI = 2) were transduced in primary neurons at DIV 2 and incubated for 72 hours prior to protein extraction.

### Mammalian cell lines and cell culture

HEK293T WT and NHE6-null HEK293T cells were cultured in Dulbecco’s modified Eagle’s medium (Invitrogen #10569044) supplied with 10 % fetal bovine serum (FBS; Invitrogen #16000044), 1 % antibiotic-antimycotic (Sigma #A8674) at 37 °C and 5 % CO_2_. The details about the cell line generation and validation for the HEK293T cells were previously described^43^.

### Live-imaging and fluorescence recovery after photobleaching (FRAP)

Primary neurons from male controls and mutants were grown on PDL-coated dishes. Seeding density are indicated above. Cells were transfected with mEmerald-Rab7 a day prior to imaging at DIV 7–9. Cells were incubated with 1 μg of plasmid DNA mixed with 2 μL of lipofectamine 2000 (Invitrogen #11668019) on 37 °C for 2 hours. After the incubation, the transfection medium was replaced with the culture medium pre-saved from the cultures prior to the transfection. On a day of imaging, primary neurons were incubated in imaging media containing phenol red-free neurobasal media (Thermo Fisher #12348017) supplemented with 2 % B27, 1 % glutamax, and penicillin/streptomycin. mEmerald-Rab7a-7 was a gift from Michael Davidson (Addgene plasmid # 54244; http://n2t.net/addgene:54244; RRID:Addgene_54244). TBC1D5 shRNA lentivirus (GeneCopoeia #LPP-RSH087343-LVRH1MP-200) was transduced at DIV 2 (MOI = 2) for 72 hours. For live-imaging, images were acquired on Olympus FV3000 confocal microscope using 60x (NA 1.42) oil immersion objective (Olympus UPlanSApo) with 2x digital zoom-in. Live cells were imaged in a temperature-controlled chamber (37 °C) at 5 % CO2 at 1 frame per second for 90 seconds. The total 4 μm of Z-stack was acquired in 0.44 μm step size. Only the neurons with identified axons were imaged. Images were deconvoluted and maximum projected in Olympus Cell Sense software. Kymographs were acquired and analyzed with “KymoAnalyzer”^107^ Plugin in Fiji ImageJ. For FRAP, region of interest (ROI) was pre-determined in the neuronal cell bodies with stationary endosomes. 3 seconds of pre-bleached images were acquired. Photobleaching was performed with 5 % of 488 nm laser for 500 milliseconds with no interval delay. After bleaching, images were live recorded for 100 seconds at 1 frame per second. Deconvoluted images were analyzed using “Stowers” Plugins in Fiji ImageJ.

### RILP assay

Brains from controls and mutants at 6–8 weeks were lysed. For mammalian cells, NHE6-null HEK293 cells were transfected using Lipofectamine 2000 (1 μg: 2 μL ratio for plasmid DNA: reagent) a day prior to the experiment. Samples were lysed in a pull-down lysis buffer (Thermo Scientific #1858601), then followed by centrifugation at 13,000 xg for 10 minutes at 4 °C. 10 % of protein lysates were saved for the input. 500 μg of protein lysates were incubated with 5 μg of His-RILP (NovoPro Labs #512098) or His-Myc using His Pull-down kit (Thermo Scientific #21277) for 2 hours at 4 °C. A separate incubation of samples with His-RILP was set up in presence of 10 μM GTPγS (NewEast Bioscience #30303), a non-hydrolysable G-protein-activating analog of GTP, or 1 mM of GDPβS (Sigma #G7637) at 30 °C for 30 minutes prior to the pull-down. Samples were then eluted with 300 mM imidazole and mixed with loading buffer and reducing agent before they were boiled at 95 °C for 5 minutes. Western blotting was performed to detect Rab7 (abcam #ab137029) from the elutants.

### GTP-Rab7 pull-down assay

This assay was adopted and modified from the Rab5-GTP pull-down assay^63,64,108,109^. Brain lysates from WT and NHE6-null rats at 6–8 weeks were centrifuged at 13,000 xg for 10 minutes at 4 °C. The supernatant was transferred to a new tube and the protein concentration was measured by BCA assay. 200 μg of protein lysates was incubated with 50 μL of GTP-agarose (Sigma #G9768) for 2 hours at 4 °C. The samples were spun down at 5,000 xg at 4 °C for 1 minute. The supernatant was discarded, and the pellet was washed with lysis buffer for 3 times. After the last wash, the samples were mixed with loading buffer and reducing agent and boiled at 95 °C for 5 minutes. Western blotting was performed to detect Rab7.

### Immunofluorescence

Primary neurons from controls and mutants were grown on PDL-coated German glass cover slip #1.5. Seeding density are indicated above. Primary neurons were washed three times with ice-chilled PBS and fixed in 3.7 % paraformaldehyde for 10 minutes. Cells were again washed with PBS for 5 minutes, three times, and permeabilized with PBS containing 0.1 % Triton-X100 (PBS-T; Sigma) for 10 minutes. Cells were blocked in blocking buffer (PBS-T containing 10 % goat serum) for one hour at room temperature. All primary antibodies except Rab7 antibodies were diluted in the blocking buffer and incubated for overnight, 4 °C. Rab7 antibodies were incubated for more than 48 hours at 4 °C. Secondary antibodies and Hoechst 33342 (Thermo Scientific #H1399) were diluted in the blocking buffer as well and incubated for one hour at room temperature. Cells were washed three times with PBS for 5 minutes for each wash and mounted with ProLong Gold Glass Antifade mountant (Invitrogen #P36984). Images were acquired on a laser-scanning confocal Nikon AX R with NSPARC detector using 60x (NA 1.42) oil immersion objective (Nikon Plan Apo λD) with 4x digital zoom-in. For NHE6-TBC1D5-Rab7 co-localization, the images were obtained with the SR mode in 0.1 μm z-step size. The pixel size was 0.04 μm for the acquisition. The images were denoised and deconvoluted in Richardson-Lucy method with 20 iterations in Nikon NIS software. For high-content imaging, the images were obtained in a spinning disk confocal CellCarrier-96 Ultra microplate (PerkinElmer). The quantification was performed in Opera Phenix Harmony (PerkinElmer) to calculate the Mander’s coefficient. The list of the primary antibodies used for immunostaining in this study: mouse anti-SMI32 (1:200, BioLegend #801701), mouse anti-NeuN (1:500, Novus #NBP1–92693), mouse anti-Tau (1:200, Millipore #MAB3420), chicken anti-MAP2 (1:500, Millipore #AB-15452), rabbit anti-NHE6 (1:500, Covance, in-house made), rabbit anti-Rab7 (1:200, abcam #ab137029), rat anti-Rab7 (1:200, BioLegend #850402), rabbit anti-Rab5 (1:200, CST #3547s), rabbit anti-TBC1D5 (1:100, Proteintech #17078–1-AP), mouse anti-TBC1D5 (1:100, Santa Cruz #sc-376296), GFP (1:100, Santa Cruz #SC9996). All the secondary antibodies in this study were IgG (H+L) highly cross-absorbed AlexaFluor secondary antibodies (1:500 dilution, Invitrogen): Goat anti-Rabbit IgG Alexa Fluor 488 (Invitrogen # A-11008), Goat anti-Mouse IgG Alexa Fluor 488 (Invitrogen # A-11001), Goat anti-Rabbit IgG Alexa Fluor 555 (Invitrogen # A-21428), Goat anti-Mouse IgG Alexa Fluor 555 (Invitrogen # A-21422), Goat anti-Chicken IgY Alexa Fluor 647 (Invitrogen # A-21449), Goat anti-Rat IgG Alexa Fluor 647 (Invitrogen # A-21247).

### Structure illumination microscopy (SIM)

Primary neurons from NHE6-null rats and their wild-type littermates were grown on #1.5 PDL-coated coverslips and fixed in 3.7 % paraformaldehyde for 10 minutes at room temperatures. The same immunofluorescence staining procedures were followed as described above. Cells was imaged on 60x oil immersion objective (NA 1.46; Olympus PlanApo N) with immersion oil (1:1 mixed two oils with refractive index 1.516 and 1.518, respectively) using the DeltaVision OMX SR imaging system (GE).

### Immunoprecipitation and western blotting

For immunoprecipitation and western blots, whole brains from WT and NHE6-null rats, or mammalian cells were lysed in ice-chilled 1 % NP-40 buffer (Invitrogen #FNN0021) with complete proteinase inhibitor cocktail (PIC; Sigma #5892970001), 10 μL/mL phenylmethylsulfonyl fluoride (PMSF; Sigma #P7626), and phosSTOP phosphatase inhibitor (Sigma #4906837001). Lysates were incubated on ice for 30 minutes and then spun down at 13,200 xg, 4 °C for 15 minutes. 10 % of the input was saved before the immunoprecipitation. For immunoprecipitation, 200 μg of samples were pre-cleared for 1 hour on end-over-end rotator with 10 μg of M-270 Epoxy Dynabeads (Life Technologies #14302D) at 4 °C. The supernatants were incubated with 10 μg of NHE6 antibody-conjugated Dynabeads on the rotator at 4 °C for overnight. After the incubation, the samples were washed three times with ice-chilled lysis buffer. Samples were mixed and boiled at 95 °C with NuPage 4× LDS sample buffer (Invitrogen #NP0007) and NuPage 10x sample reducing agent (Invitrogen #NP0009). The samples were run on NuPage 4–12 % SDS-PAGE gel (Invitrogen #NP0321BOX) using MOPS buffer and transferred to nitrocellulose membranes. After three washes with TBS containing Tween20 (TBS-T), IRDye 680W and 800W goat anti-rabbit and anti-mouse secondary antibodies were incubated with samples for 1 hour at room temperature. All the membranes were visualized with the LiCor Odyssey Clx Infrared Imaging System. The list of the primary antibodies was used in this study: Rabbit anti-NHE6 (1:1000, Covance, in-house made), rabbit anti-TBC1D5 (1:1000, Novus Biological # NBP1–93653), rabbit anti-Rab7 (1:1000, abcam #ab137029), rabbit anti-HA (1:1000, CST #3724s), mouse anti-Myc (1:1000, Santa Cruz #sc-40), mouse anti-actin (1:5000, Proteintech #66009–1-Ig), rabbit anti-RILP (1:1000, Genetex #GTx32069), mouse anti-GAPDH (1:5000, #G8795) and rabbit anti-vinculin (1:1000, CST #4650) were used.

### Endosomal pH measurement

Primary neurons were seeded at densities of 28,000 cells per well on CellCarrier-96 Ultra microplate (PerkinElmer). The assay was conducted as previously described in detail^69^. Briefly, primary neurons at DIV 7 were first starved in EBSS at 37 °C for 30 minutes. EBSS was then replaced with Neurobasal A media with 33 μg/ml fluorescein isothiocyanate (FITC)-conjugated transferrin (Thermo Fisher Scientific #T2871), which is pH sensitive, and 33 μg/ml Alexa Fluor 546-conjugated transferrin (Thermo Fisher Scientific # 23364), which is pH insensitive. After 10 minute-incubation, the cells were washed twice with warm PBS, placed in phenol red-free Neurobasal media, and imaged live under 60x water objective using an Opera Phenix High-Content Screening System. To generate the standard curve for use in determining endosomal pH, similar procedures were followed; however, neurons were imaged in standard buffer solutions as described previously^69^.

For late endosomal pH measurement, primary hippocampal neurons from WT and NHE6-null rat brains were transduced at DIV 2 with CellLight^™^ late endosome (Rab7)-RFP (Thermo Fischer # C10589, 30 particles per cell). At DIV 4, neurons were incubated with 500 μg/ml of Oregon green-dextran (Invitrogen #D717–0), which is pH sensitive, 500 μg/ml of Alexa647-dextran (Invitrogen # D22914), which is pH insensitive along with Hoechst 33342 (1:5000) for 1 hour at 37 °C. After the incubation, the cells were washed twice with warm 1X PBS, placed in phenol red-free Neurobasal media, and imaged live under 60x water objective using an Opera Phenix High-Content Screening System.

### Endosome-lysosome fusion assay

Primary neurons were seeded at densities of 28,000 cells per well on CellCarrier-96 Ultra microplates (PerkinElmer). The assay was conducted as previously described in detail^25,110^.

### Measurement of the catalytic activity of TBC1D5

#### Constructs.

Constructs corresponding to residues 5–176 of mouse Rab7 (NM_009005) and 15–449 of worm TBC1D5 (NM_065578; also known as RBG-3) were amplified and inserted into BamHI/SalI sites of pET15 modified to incorporate an N-terminal 6xHis tag (MGHHHHHHGS).

#### Expression and Purification.

Constructs transformed into BL21(DE3)-RIPL cells (Novagen) were expressed in 2xYT media supplemented with 100 mg/L ampicillin. After growth at 37 °C to OD_600_ ~0.2, the temperature was lowered to 22 °C until OD_600_ ~0.4. Expression was induced with 0.05 mM IPTG for 16 hours. Cell pellets were resuspended in 50 mM Tris, pH 8.0, 150 mM NaCl, 2 mM MgCl_2_, 0.1 % 2-mercaptoethanol, 0.1 mM PMSF, 0.01 mg/mL protease free DNAse I (Worthington), and 0.2 mg/mL lysozyme. After incubation on ice for 1 hour followed by sonication, the lysates were supplemented with 0.5 % Triton X-100 and centrifuged at 28,000 ×g for 1 hour at 4 °C. After purification over NiNTA-sepharose HP, HiTrap QHP and Superdex 200 columns, proteins were estimated to be >95 % pure by SDS-PAGE. For mutation experiments, RBG-3 H166 (corresponding to H207 of human TBC1D5) was mutated to asparagine or serine in the WT constructs (Genescript). The expression and purification were identical to the WT constructs.

#### Nucleotide Loading.

Rab7 (1 mg/mL) was incubated for 2 hours at room temperature with a 20-fold molar excess of GTP in 20 mM Tris, pH 7.5, 100 mM NaCl, 5 mM EDTA, and 1 mM DTT. Excess nucleotide was removed using a 10 mL D-Salt column (Thermo-Fisher) equilibrated with 20 mM Tris, pH 7.5 and 150 mM NaCl.

#### GAP Assays.

Single turnover GAP assays were performed as described^111^, using the increase in intrinsic tryptophan fluorescence of Rab7 to monitor the rapid conformational switch from the GTP- to GDP-bound state resulting from GTP hydrolysis^18^. Reactions were initiated by mixing 50 μL of 4 μM Rab7-GTP in 20 mM buffer, 150 mM NaCl with an equal volume of the same buffer containing 10 mM MgCl_2_ and 0 – 2.56 μM RBG-3. Buffers were sodium acetate (pH 4.5–5.5), PIPES (pH 6.0–7.0), and Tris (pH 7.5–8.5). We observed aggregation of RBG-3 H166S and H166N at pH 5.0 or below. Dispensing, mixing and dilutions were performed with a Precision 2000 automated multichannel pipetting system (Biotek). The increase in Rab7 intrinsic tryptophan fluorescence accompanying GTP hydrolysis was continuously monitored in half area microplates (Corning) using a Tecan Spark microplate spectrometer with excitation at 290 nM, emission at 340 nm, and a bandwidth of 10 nM. Fluorescence time courses F_t_ were corrected for a small amount of photobleaching and catalytic efficiencies obtained directly from simultaneous fits with the global exponential model function

$${\text{F}}_{\text{t}}={(\text{F}}_{0}-{\text{F}}_{\infty })\text{exp}({-\text{k}}_{\text{obs}}\;\text{t})+{\text{F}}_{\infty }$$

F_0_ and F_∞_ are the fluorescence intensities at t = 0 and t→∞, respectively, and k_obs_ is calculated at each [GAP] as:

$${\text{k}}_{\text{obs}}={(\text{k}}_{\text{cat}}/{\text{K}}_{\text{M}})[\text{GAP}]+{\text{k}}_{\text{intr}}$$

where k_intr_ is the intrinsic rate constant for GTP hydrolysis in the absence of a GAP. For the simultaneous fits, k_cat_/K_M_ and k_intr_ were treated as global parameters for all [GAP] whereas F_0_ and F∞ were treated as independent local parameters for each [GAP]. In some cases, a linear term was included to account for a small increase or decrease in fluorescence observed at higher GAP concentrations. The pH dependence of the catalytic efficiency was fit with the model function:

$${\text{k}}_{\text{cat}}/{\text{K}}_{\text{M}}(\text{pH})=({\text{k}}_{\text{cat}}/{\text{K}}_{\text{M}}(\infty )-{\text{k}}_{\text{cat}}/{\text{K}}_{\text{M}}(0))/(1+10^{\text{pKa}-\text{pH}})+{\text{k}}_{\text{cat}}/{\text{K}}_{\text{M}}(0)$$

where k_cat_/K_M_(pH) is the observed rate constant as a function of pH and k_cat_/K_M_(0) and k_cat_/K_M_(∞) are the fluorescence intensities at pH → 0 and pH → ∞, respectively. This model describes the pH dependence for one or more titrating groups with a common pK_a_ such that k_cat_/K_M_(0) corresponds to the catalytic efficiency of the fully protonated state and k_cat_/K_M_(∞) to that of the fully unprotonated state. For fitting the WT data, k_cat_/K_M_ and k_cat_/K_M_(∞) were treated as adjustable parameters whereas k_cat_/K_M_(0) was fixed at zero since fitting it resulted in a small, nonphysical negative value. For H166N and H166S, k_cat_/K_M_, k_cat_/K_M_(∞) and k_cat_/K_M_(0) were treated as adjustable.

#### Statistical assessment of parameter uncertainty.

To quantitatively assess the uncertainties related to the maximum likelihood parameter values in the fitted model for the pH dependence of the mutant vs. WT proteins, two ‘gold standard’ approaches were used [Press, W. H., Teukolsky, S. A., Vetterling, W. T., & Flannery, B. P. (2007). *Numerical Recipes: The Art of Scientific Computing* (3rd ed.). Cambridge University Press]. This rigorous statistical assessment was also used to address the extent to which the maximum likelihood parameter values are affected by fitting the WT data over the same pH range as the mutants.

First, the χ^2^ merit function was mapped for each parameter over a range of fixed values while fitting the remaining parameters to determine χ^2^ for each value of the fixed parameter. Noting that χ^2^ is the negative log of the corresponding likelihood probability density function (PDF) that the data would be observed given a particular instance of parameter values, the χ^2^ values were inverse transformed to probability densities as exp(-χ^2^) and normalized such that the cumulative PDF has a maximum value of 1. The resulting probability densities are well described by Gaussian (i.e., Normal) functions with clearly defined maxima (see Extended Fig. 6c). These observations are consistent with the maximum likelihood assumption, and further justify the χ^2^-mapping and interpretation described here. Though important, the results of this analysis apply to the particular instance of noise present in the experimentally determined k_cat_/K_M_ values. Consequently, it can be anticipated that different sets of parameter values would be observed for different instances of noise. To assess the impact of normally distributed variation in measurement errors on the fitted parameter values, 100 Monte Carlo synthetic data sets were generated by combining the maximum likelihood models calculated at the same pH values as the experimental data with different instances of Gaussian noise having a standard deviation equal to that of the relevant experimental data. These Monte Carlo synthetic data sets were fit with the same model function used to analyze the experimental data to determine maximum likelihood parameter values for each synthetic data set (see Extended Data Fig. 6d).

Both approaches demonstrate clear differences between the constant (k_cat_/K_M_(0)) and amplitude (k_cat_/K_M_(∞) - k_cat_/K_M_(0)) values for the mutants vs. WT that are substantially greater the differences in 99.7% confidence intervals (equivalent to ± 3 SDs). However, given that the differences in pK_a_ values are evidently less significant (only modestly greater than the 68.3 CI equivalent to ± 1 SD), it is relevant to consider the possibility that the pK_a_ values for the mutants and WT are the same and that the observed differences in the fitted values are a consequence of measurement errors. To examine this possibility, the experimental data for all three proteins were simultaneously fit with a pH dependence model in which the pK_a_ was treated as a global parameter whereas the constant and amplitude were treated as local parameters (see Extended Data Figure. 6e). This approach results in a common pK_a_ value equivalent to that previously observed for the WT protein as expected, with modest decreases in the differences between the constant and amplitude values for the mutant vs. WT proteins. Nevertheless, the differences in the constant and amplitude values remain substantially larger than the 99.7% confidence intervals (equivalent to ± 3 SDs). Consistent with this observation, using global values for the constant and/or amplitude and a local value for the pK_a_ results in large increases in χ^2^ that are highly improbable.

#### Software.

The Mac OSX application DELA was used for data processing, analysis and relevant figures^111,112^. Associated Python 3 scripts were used for extension of program functionality as well as control and automation.

### AlphaFold 3 structural modeling

The AlphaFold 3 structural model for the dimeric NHE6-TBC1D5-Rab7 complex was generated using the AlphaFold 3 server^70^, with two copies of the amino acid sequences for human NHE6 (NP_001036002; Uniprot Q92581–2), human TBC1D5 (NP_001127853; Uniprot Q92609–2), and human Rab7 (NP_004628), two copies of GTP, and two copies of Mg^2+^. Figures for the model were rendered with PyMOL.

### Phosphate assay

The release of phosphate was measured using EnzCheck Phosphate Assay kit (Invitrogen #E6646) following the manufacturer’s protocol. GTP was pre-loaded to Rab7 for 2 hours at room temperature. GTP-Rab7 was incubated with 67.5 nM of TBC1D5 (Origene, #TP30440), and 0–400 nM of NHE6 (abcam # 161011).

### Mathematical modeling for local pH and proton concentration

Please see the Supplementary Equation for the full analysis.

### Electron microscopy (EM)

Genotypes and conditions of animals were blinded during image collection and analyses. Tissue was collected and processed for standard electron microscopic analysis as previously described^113^.

### Yeast two hybrid

The coding sequences of NHE6 and TBC1D5 proteins were cloned into pGBKT7 (bait) and pGADT7 (prey) vectors, respectively. The constructs were co-transformed into Y2HGold yeast strains using the standard lithium acetate (LiAc) transformation method. Transformed yeast were plated on YPDA plates (1 % yeast extract, 2 % tryptone, 2 % glucose, 0.02 % adenine) and incubated at 30 °C for 2–3 days to allow colony formation. Individual colonies were picked and grown overnight in YPDA medium at 30 °C with shaking at 230–250 rpm for 16–20 hours. Yeast cells were harvested by centrifugation at 700 × g for 5 minutes at room temperature, and resuspended in 1.1× TE/LiAc solution (prepared by mixing 1.1 mL of 10× TE buffer with 1.1 mL of 1 M LiAc, then bringing to the desired final volume with ddH_2_O). The reactions were set up for positive control (pGBKT7-p53+pGADT7-T), negative control (pGBKT7-lam+pGADT7-T), bait #1 and bait #2. Cells were plated on synthetic defined (SD) dropout media to assess interaction: SD medium without Trp and Leu (for transformation selection), SD medium without Trp, Leu, and His (for moderate stringency interaction selection), and SD medium without Trp, Leu, His, and Ade (for high stringency interaction selection).

Bait and prey peptide sequence used for this study:

Bait #1: NHE6, 345–371 a.a. TALVTKFTKLREFQLLETGLFFLMSWS

Bait #2: NHE6, 536–701 a.a.

LHIRVGVDSDQEHLGVPENERRTTKAESAWLFRMWYNFDHNYLKPLLTHSGPPLTTTLPACCGPIARCLTSPQAYENQEQLKDDDSDLILNDGDISLTYGDSTVNTEPATSSAPRRFMGNSSEDALDRELAFGDHELVIRGTRLVLPMDDSEPPLNLLDNTRHGPA

Prey: TBC1D5, full length

MYHSLSETRHPLQPEEQEVGIDPLSSYSNKSGGDSNKNGRRTSSTLDSEGTFNSYRKEWEELFVNNNYLATIRQKGINGQLRSSRFRSICWKLFLCVLPQDKSQWISRIEELRAWYSNIKEIHITNPRKVVGQQDLMINNPLSQDEGSLWNKFFQDKELRSMIEQDVKRTFPEMQFFQQENVRKILTDVLFCYARENEQLLYKQGMHELLAPIVFVLHCDHQAFLHASESAQPSEEMKTVLNPEYLEHDAYAVFSQLMETAEPWFSTFEHDGQKGKETLMTPIPFARPQDLGPTIAIVTKVNQIQDHLLKKHDIELYMHLNRLEIAPQIYGLRWVRLLFGREFPLQDLLVVWDALFADGLSLGLVDYIFVAMLLYIRDALISSNYQTCLGLLMHYPFIGDVHSLILKALFLRDPKRNPRPVTYQFHPNLDYYKARGADLMNKSRTNAKGAPLNINKVSNSLINFGRKLISPAMAPGSAGGPVPGGNSSSSSSVVIPTRTSAEAPSHHLQQQQQQQRLMKSESMPVQLNKGDVVTGSDAQVSVPVQTLTDLQGLSSKNISSSPSVESLPGGREFTGSPPSSATKKDSFFSNISRSRSHSKTMGRKESEEELEAQISFLQGQLNDLDAMCKYCAKVMDTHLVNIQDVILQENLEKEDQILVSLAGLKQIKDILKGSLRFNQSQLEAEENEQITIADNHYCSSGQGQGRGQGQSVQMSGAIKQASSETPGCTDRGNSDDFILISKDDDGSSARGSFSGQAQPLRTLRSTSGKSQAPVCSPLVFSDPLMGPASASSSNPSSSPDDDSSKDSGFTIVSPLDI

### Quantification and Statistical Analysis

All statistical methods and number of biological replicates used for this study are described in the figure legends and the Methods. In this study, values from each biological replicate are clustered in different color codes and plotted as a small dot. Means from each biological replicate are overlaid on the top of the full dataset as a bigger dot. All the statistical analyses were calculated across biological replicates (represented as bigger dots), not the entire dataset (represented as small dots)^114^. Data are represented as means ± SEM throughout the manuscript. Asterisks represent p-value as follows: **** < 0.0001, *** < 0.001, ** < 0.01, and * < 0.05.

## Extended Data

**Figure:**
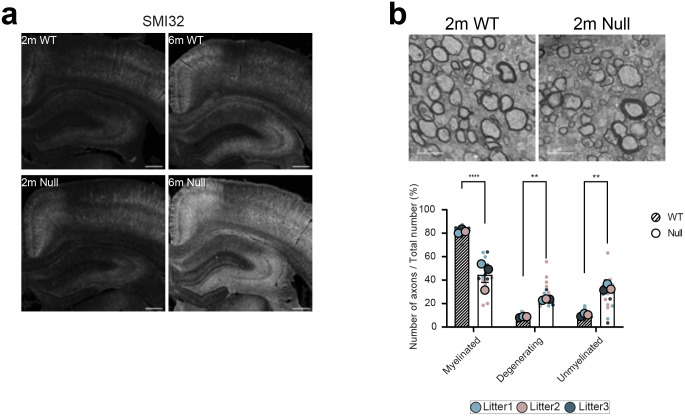


**Figure:**
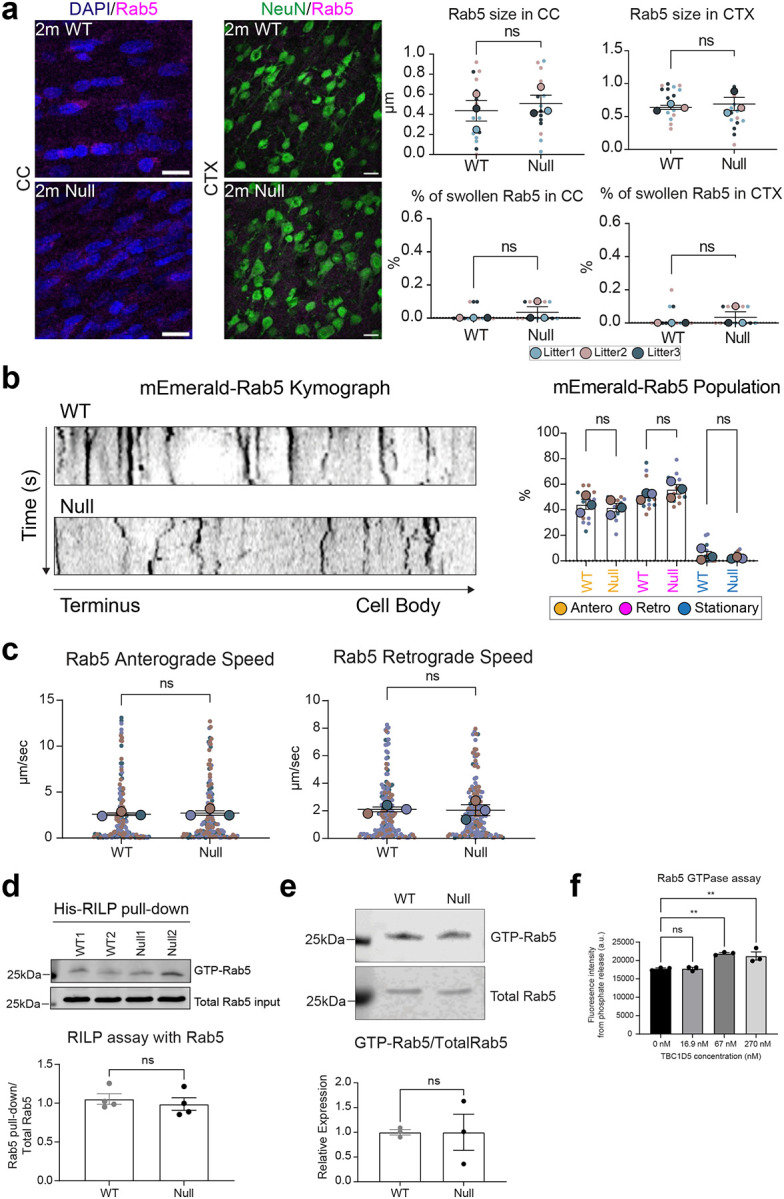


**Figure:**
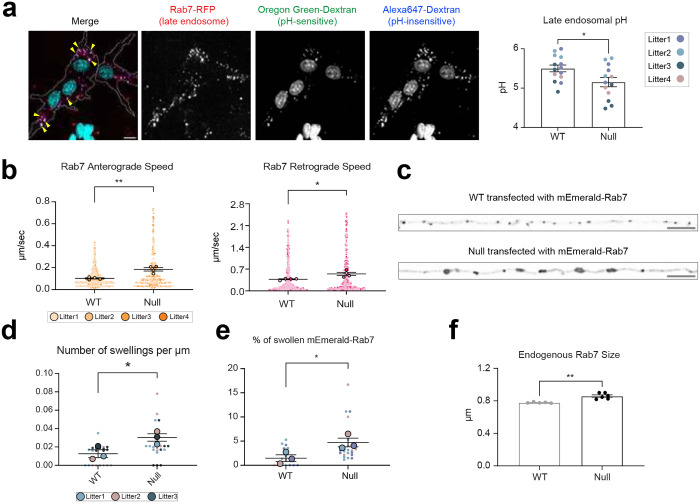


**Figure:**
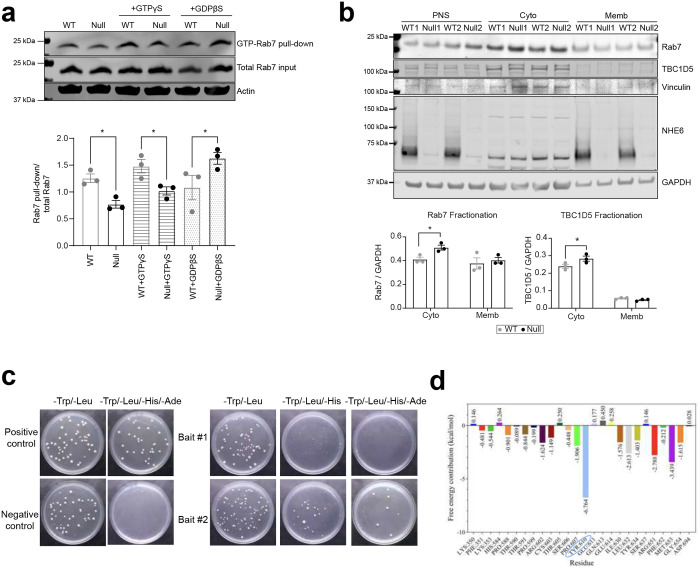


**Figure:**
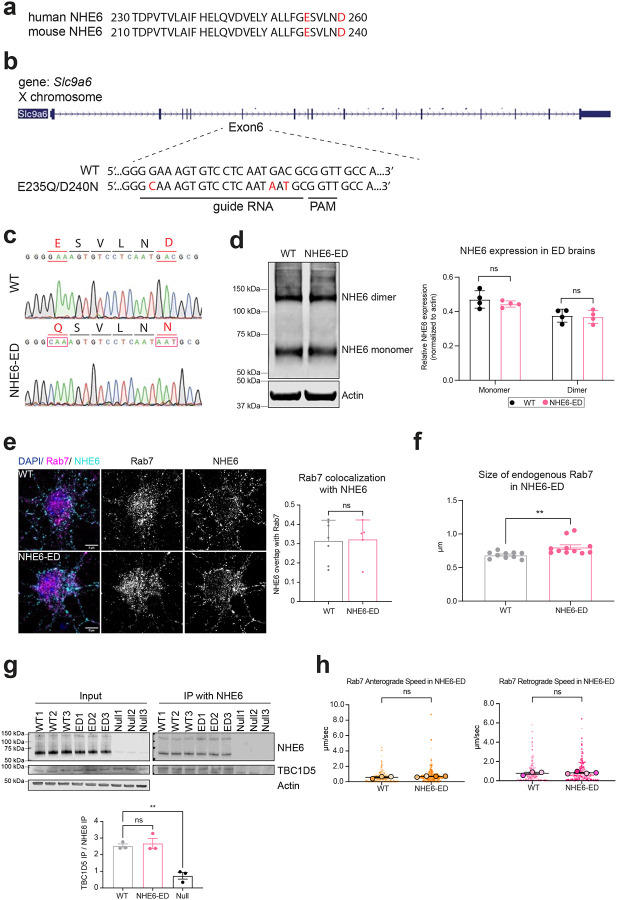


**Figure:**
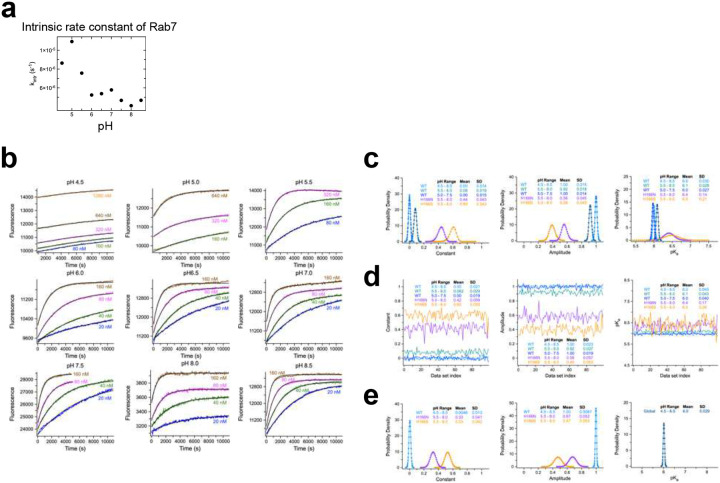


**Figure:**
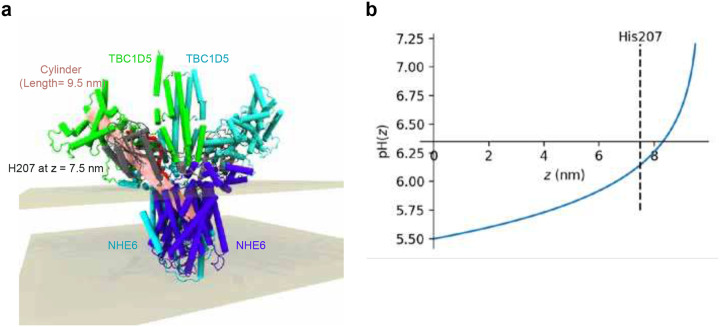


**Figure:**
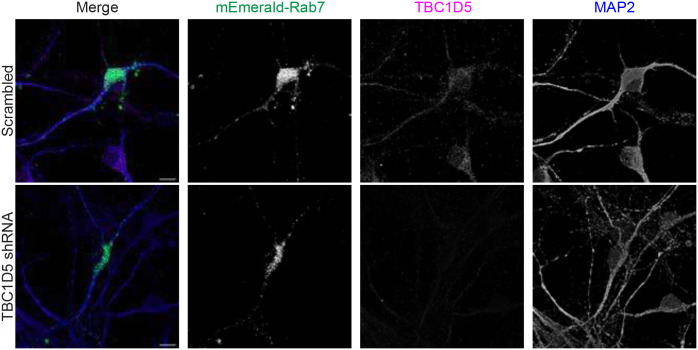


**Figure:**
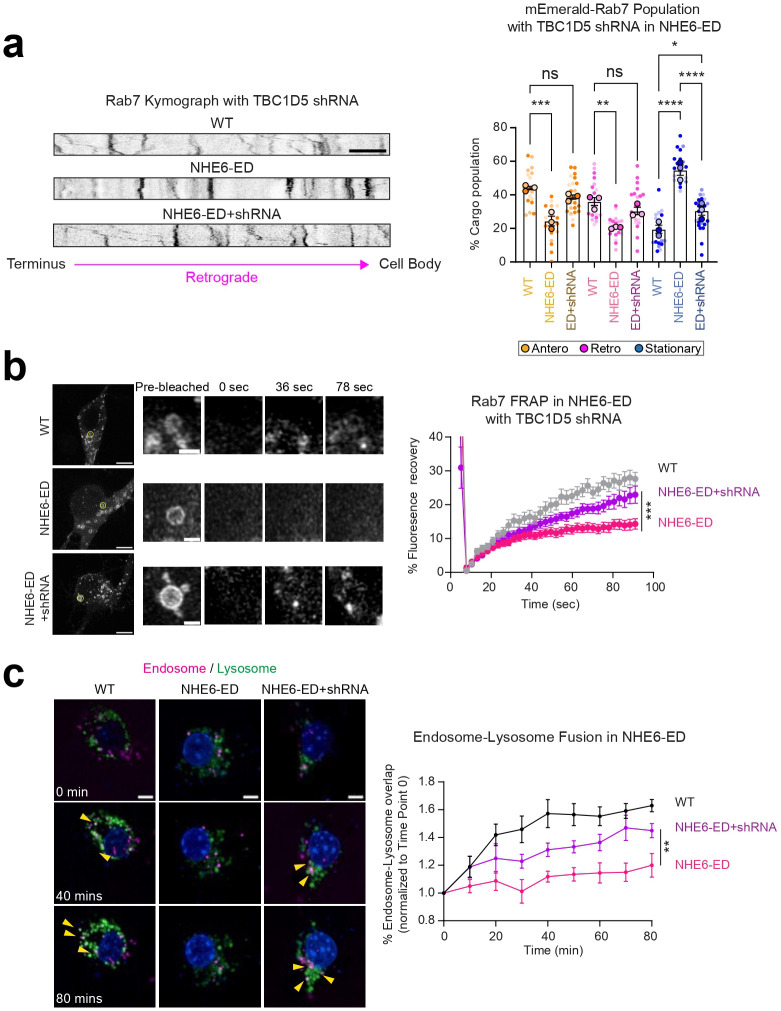


## Supplementary Material

Supplementary Files

This is a list of supplementary files associated with this preprint. Click to download.

- LeeetalExtendedFiguresLegends.pdf
- LeeetalSupplementaryEquation.pdf

## Data Availability

Plasmids, animals, and cell lines generated in this study are available upon request. We generated a new transgenic mouse line (NHE6-ED) for this study. All data reported and any additional information required for reanalysis of the data in this paper will be shared by lead contact upon request.

## Code Availability

Python scripts used for Alpha Fold 3 structure modeling, statistical analyses, and computational analyses are available on Github (https://github.com/Becksteinlab/TBC1D5-Rab7-NHE6-proton-diffusion-model). All data reported and any additional information required for reanalysis of the data in this paper will be shared by lead contact upon request.
